# The absence of canonical respiratory complex I subunits in male-type mitogenomes of three *Donax* species

**DOI:** 10.1038/s41598-024-63764-8

**Published:** 2024-06-24

**Authors:** Artur Burzyński, Beata Śmietanka, Jenyfer Fernández-Pérez, Marek Lubośny

**Affiliations:** 1 Department of Genetics and Marine Biotechnology, Institute of Oceanology Polish Academy of Sciences, Sopot, Poland; 2 Departamento de Bioloxía, Facultade de Ciencias and CICA (Centro de Investigacións Científicas Avanzadas), Universidade da Coruña, Campus de A Zapateira, A Coruña, Spain

**Keywords:** mtDNA, NGS, Bivalvia, DUI, NADH, Doubly uniparental inheritance, Marine biology, Next-generation sequencing, Evolutionary biology, Evolutionary genetics, Taxonomy, Mitochondria

## Abstract

Bivalves are an extraordinary class of animals in which species with a doubly uniparental inheritance (DUI) of mitochondrial DNA have been described. DUI is characterized as a mitochondrial homoplasmy of females and heteroplasmy of male individuals where F-type mitogenomes are passed to the progeny with mother egg cells and divergent M-type mitogenomes are inherited with fathers sperm cells. However, in most cases only male individuals retain divergent mitogenome inherited with spermatozoa. Additionally, in many of bivalves, unique mitochondrial features, like additional genes, gene duplication, gene extensions, mitochondrial introns, and recombination, were observed. In this study, we sequenced and assembled male-type mitogenomes of three *Donax* species. Comparative analysis of mitochondrial sequences revealed a lack of all seven NADH dehydrogenase subunits as well as the presence of three long additional open reading frames lacking identifiable homology to any of the existing genes.

## Introduction

There are different molecular markers which complement our knowledge concerning the level of population variability or even evolutionary history of whole species. Mitochondrial DNA (mtDNA) is used by researchers as a neutral or nearly neutral marker useful for population, phylogeographic and phylogenetic analyses. Until recently, the strict maternal inheritance (SMI) of the mtDNA paradigm has been the most common in most Metazoan species^1–3^. Under this mode, the mitochondrial genome is transmitted to the progeny exclusively through the female lineage. One of the alternative inheritance modes, termed as doubly uniparental inheritance (DUI), has been discovered in some bivalves lineages^4–6^. Under DUI, females pass their mitogenome (F genome) to both offspring, whereas males possess an additional mtDNA (M genome) which is transmitted nearly exclusively to their sons. Thus, in male specimens F mitogenome predominates in somatic tissues whereas M mitogenome is normally prevalent in gonads. For this reason, females are homoplasmic, while males are heteroplasmic, possessing two different mitogenomes inherited from both parents. In detail the whole DUI phenomenon becomes a bit more intricate due to reports of exceptions form the general rule, such as the leakage of M-type mitogenomes to somatic tissues^7–10^, the identification of M-type mtDNA in female individuals^11–13^, masculinization^14–16^, recombination^17,18^, or even instances of heteroplasmy not correlated with sex^19^. Mitochondrial M and F lineages evolve at different rates. The faster evolution of the M genome is associated with the more relaxed selection and can contribute to high M *vs* F mtDNA sequence divergence, reaching even up to 52% (uncorrected amino acid p-distance) as seen in the example of *Quadrula quadrula* (Unionidae)^20^. Both M and F lineages can unveil entirely different population structures for compared data, as it was observed in European *Mytilus* mussels^21^. The DUI phenomenon was initially discovered in *Mytilus edulis* mussels, but it has since been confirmed in many other bivalvian species^16^. This mode of mtDNA inheritance is also supposedly present in wedge shellfish *Donax trunculus*^22^.

The wedge edible selfish of the genus *Donax* (Bivalvia, Donacidae) is a very important, numerous, and dominant component of the sandy beaches environment macrofauna in tropical, subtropical, and temperate climate zones^23^. There are five sympatric European *Donax* species in the littoral waters surrounding the Iberian Peninsula: *D. trunculus* (Linnaeus, 1758), *D. vittatus* (Da Costa, 1778), *D. variegatus* (Gmelin, 1791), *D. semistriatus* (Poli, 1775) and sporadically found *D. venustus* (Poli, 1775). Some of those species are commercially important due to their high exploitation for consumption purposes in many European countries but some are locally consumed or harvested as fishing bait. In some cases, the intense fishing harvesting has led to overexploitation, as it has been observed in Galicia for *D. trunculus* – species intensively exploited in France, Spain, Portugal, Italy, and Turkey^24–28^. Also in some southern and Mediterranean coasts of the Iberian Peninsula natural beds of *D. trunculus* seem to be at a high long-term risk of extinction^29^. *Donax trunculus* as a species with greater commercial value, in its shape, size and colour is similar to other *Donax* species which are not so economically valued but might be marketed together^30^. Thus, protection and support of natural variable genetic resources of *Donax* genus seems to be very important for ecological but also for economic aspects. At the time of writing, complete sequences of female mitogenomes are available for four species of *Donax* from the Iberian Peninsula.^30^.

Here we ascertain, for the first time, the complete male mitochondrial genome sequences obtained for three *Donax* species: *D. trunculus*, *D. vittatus* and *D. semistriatus* from the Iberian Peninsula.

## Results

**Figure 1:**
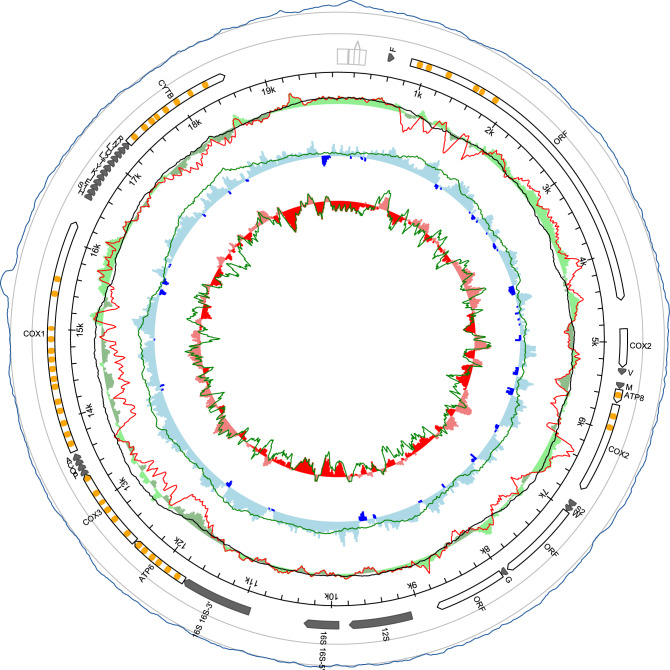
Genetic map of the M-type *Donax vittatus* mitogenome. The presented features follow the convention of mitoconstricor (https://github.com/aburzynski/mitoconstrictor). Outer blue line represents sequence coverage with sequencing reads (grey circles 10 × and 100 × coverage). Arrows represent identified genes, tRNAs and open reading frames. Orange ovals on arrows represent identified transmembrane domains. Transparent light-gray boxes represent repetitive sequences in non-coding region. Sequence local compositional bias is represented by inner circles, calculated in a 200 bp long sliding window with 25 bp steps. First inner green circle represents local AT-skew; blue circle represents local GC-skew; red circle represents local GC content relative to average of the whole mitogenome. Red line represents filtered AT-skew at non-coding regions, and second codon position. Black and two green lines represent filtered AT-skew, GC-skew and GC content respectively, at neutral sites, calculated in a 1.000 bp long sliding window. Genetic maps for all three mitogenomes with cartoon legend can be found in supplementary data.

**Figure 2:**
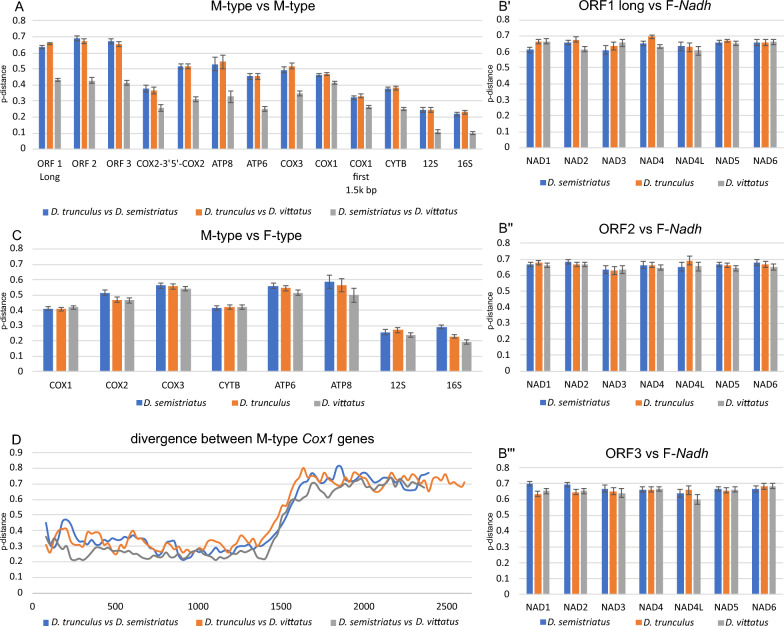
Divergence (p-distance) between *Donax* spp. genes. (**A**) Divergence between mitochondrial genes for M-type *Donax* pairs. (**B**’–**B**’’’) Divergence between three ORFs from M-type *Donax* spp. mtDNA and *nadh* genes from F-type mtDNA. (**C**) Divergence for mitochondrial gene pairs between M-type and F-type *Donax* mitogenomes. (**D**) Sliding widow divergence analysis for M-type *cox1* gene pairs (100 bp window, 25 bp step).

In this study, we were able to assemble (NOVOplasty^31^ and CLC genomics workbench) and annotate (MITOCONSTRICTOR) 3 male-type (M) mitogenomes from *Donax* genus (Fig. 1). The size of mtDNAs varied form 19,088 bp in *D. semistriatus*, 19,858 bp in *D. vittatus* to 20,628 bp in *D. trunculus*. All of them encoded six protein-coding genes (*cox1*-*cox3*, *cytb*, *atp6*, *atp8*), two rRNA subunits, and twenty-two tRNAs. The identification was performed through Blast^32^, Wise2^33^, ARWEN^34^, and Infernal^35^ searches based on various sequence profiles and databases. None of the seven complex I (NADH dehydrogenase) subunit genes were identified, despite the presence of three long unassigned open reading frames in all mitogenomes. In theory, these ORFs were the most suitable candidates to encode the missing NADH subunits (length of ORF1 3675, 3714 and 4146 bp, ORF2 921, 915 and 996 bp, ORF3 801, 798 and 972 bp). The divergence (nucleotide p-distance) of those ORFs between sequenced M-type mitogenomes was very high (0.414–0.689), indicating some homology between the closest related sequences of *D. semistriatus* and *D. vittatus* only (Fig. 2A)*.* However, attempt to align those ORF with any of the possible *nadh* subunits genes from female-type *Donax* mitogenomes gave inconclusive results (Fig. 2B, p-distance 0.601–0.699). None of the *nadh vs* ORF pairs exhibited a noticeable decrease in p-distance that could be definitively interpreted as homology between the respective genes or proteins (Fig. 2C)^32^. At distances exceeding 0.6, it is often challenging to determine whether homologies between codons or amino acids are genuine or if we are dealing with random noise caused by sequence aligning tools. Genes, ORFs, and proteins were aligned in MEGA^36^ using comprehensive pairings with varying gap penalties to unveil potential conserved regions possibly overlooked by Blast or Wise2. In true philosophical manner it is impossible to provide evidence of absence but with the p-distance values presented above (Fig. 2) we tried to numerically present the low probability of homology between ORFs and *nadh* sequences. Additionally, at the end of the supplementary data, we have added similar results calculated with the Tamura 3-parameter model correcting compared sequences for bias in GC-content, as well as provided graphs showing how divergence values of protein sequence pairs look versus randomised amino acid sequences generated from compared sequences (supplementary data).

In addition to unassigned ORFs, all *cox1* genes were elongated in comparison to an average length for superfamily Tellinoidea (~ 1700 bp) reaching 2391, 2607, 2748 bp Annotated *cox1* genes were losing their similarity after around the first 1500 bp (Fig. 2D) and the predicted amino acid sequence of their 3’ extensions did not match any known protein deposited in databases (GenBank, Pfam, InterPro, UniProt). Structural analysis of the elongated cytochrome oxidase subunit I did not provide any conclusive results either (supplementary Figs. 11–16). The last two unusual features of male *Donax* mitogenomes were: the *cox2* gene was split and its 5’-end was relocated, and the 16S rRNA gene was also split into two parts (Fig. 1). In the *D. trunculus* the size of a gene coding the large ribosomal subunit was close to standard (1363 bp), in *D. semistriatus* the gene was 1647 bp long with a possible 200–300 bp insertion, and in *D. vittatus* Infernal^35^ software predicted this gene in two parts with 500–600 bp long noncoding space between them (1845 bp long, if we were to assume it is one continuous gene).

**Figure 3:**
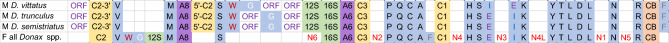
Gene rearrangement in *Donax* mitogenomes. Different color boxes represent different gene sets (rRNA and tRNA) or complexes (Complex I, III, IV and V). Changes in colors of letters were made to better visualize differences in tRNA locations.

The comparison of gene order between M and F-type mitogenomes (Fig. 3), reveals decent level of similarity. Despite the absence of all *nadh* genes, presence of additional ORFs, split of the *cox2* gene, and a few rearrangements, the order of most *trn* genes remains unaffected. This suggests limited involvement of the tandem duplication random loss (TDRL) model in the evolutionary path from F to M mitogenomes in *Donax*. Typically, TDRL processes result in frequent *trn* relocations^37,38^, but here, only three steps explain the differences. Firstly, a block of tRNA-Trp, tRNA-Gly, 12S rRNA shifted in M-type mitogenomes from between tRNA-Val and tRNA-Met to the region between tRNA-Ser and 16S rRNA, interlaced with two of the additional open reading frames. Secondly, tRNA-Phe moved from between tRNA-Ala and *cox1* to the region between *cytb* and the longest of additional ORF. Lastly, in *D. vittatus* M mitogenome only*,* tRNA-Gly switched places with one of the smaller ORFs, and tRNA-Ile switched places with tRNA-Glu.

**Figure 4:**
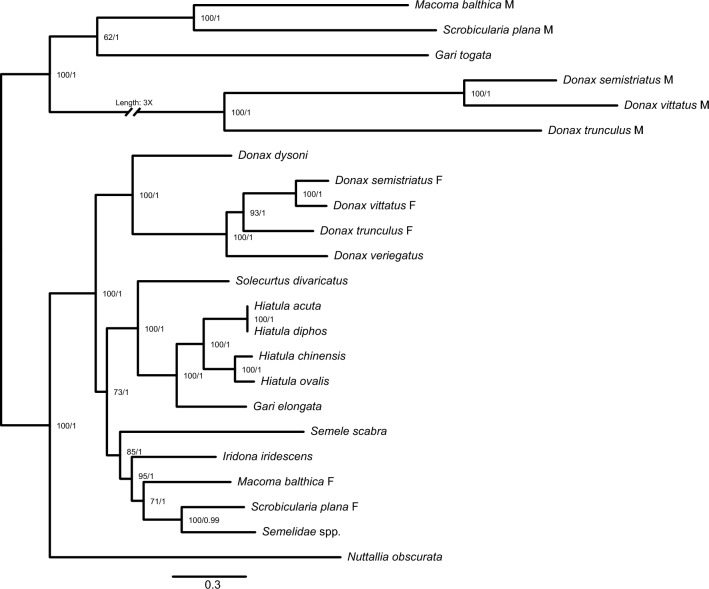
Phylogenetic reconstruction based on complete mitogenomes for superfamily Tellinoidea. Left node values (62–100) represent bootstrap values for IqTree2 Maximum-likelihood method, right node values (0.99–1) represent posterior probabilities from Beast2 (Bayesian phylogenetic analysis). GenBank accession numbers for mitogenomic sequences: *Macoma balthica* M/F—MH285593/MH285592^42^; *Scrobicularia plana* M/F – MN528026/MN528027^43^; *Gari togata*—MN164429, *Gari elongata*—MG978992, *Hiatula acuta*—MG978991, *Hiatula chinensis*—MG978990, and *Hiatula ovalis*—MG978993^41^; *Donax dysoni*—MZ362260; *Nuttallia obscurata*—JN398364, *Iridona iridescens*—JN398362, *Semele scabra*—JN398365, *Hiatula diphos*—JN398363, and *Solecurtus divaricatus*—JN398367^44^; *Semelidae* spp. —KX815956^45^; *Donax veriegatus*—KY780365, *Donax semistriatus* F—KY780363, *Donax vittatus* F—KY780366, and *Donax trunculus* F—KY780364^30^; *Donax semistriatus* M—OR416184, *Donax vittatus* M—OR416182, and *Donax trunculus* M—OR416183 (this study). Species names were updated on 22th Sep 2022 based on WoRMS database (World Register of Marine Species).

To better understand the relations of assembled M-type mitogenomes within superfamily Tellinoidea we performed phylogenetic reconstruction with two parallel methods Bayesian’s in Beast2^39^ and Maximum Likelihood in IqTree2^40^. Results from both methods gave identical topologies, due to that the acquired posterior probability values from Beast2 has been applied to the consensus tree generated with IqTree2 (Fig. 4). The phylogenetic reconstruction based on 23 complete mitogenomes showed a distinct separation into two groups consisting of only M-type, and only F-type mitogenomes. The relationship between assembled male *Donax* mitogenomes correctly reflected the configuration observed in female-type mitogenomes acquired from GenBank database. Even though it is not mentioned by original authors^41^, based on the phylogenetic tree we can also presume that the mtDNA for *Gari togata* is an M-type (the other *Gari* spp. mitogenome is clustering together with F-type mitogenomes) as well as point out that records for *Hiatula acuta* and *Soletelina/Hiatula diphos* mitogenomes are identical and probably belong to the same species (100% identity).

## Discussion

The presence of complex I genes in male-type *Donax* mitogenomes is unsure. On the one hand those additional ORFs could represent mutated and/or combined *nadh* genes. However, this is unlikely, given the lack of transmembrane domains typically found in abundance among mitochondrial *nadh* subunits (TMHMM predictions^46^, Fig. 1 and supplementary data Fig. 4 for visual comparison). Unfortunately, the function of these ORFs cannot be determined without proper proteomic studies, verifying at least co-localization of protein products of these ORFs with NADH: ubiquinone oxidoreductase enzyme and nuclear subunits within it. On the other hand, we should consider if *nadh* subunits from M-type mitogenomes are even necessary^47^. It has been shown, that human cells without mitochondria ρ^0^ can exist and even proliferate^48^. Complex I is major but not the only point of electron entry to the respiratory chain^49^, which might be limiting overall respiration rate but do not prevent it. Potentially it would only reduce oxidative phosphorylation rate of mitochondria at the same time reducing number of reactive oxygen species generated by NADH dehydrogenase^50^. This lack of *nadh* might be in line with the metabolic remodelling theory^51^, showing low oxidative phosphorylation rates in relation to the maximum electron transport system (OXPHOS/ETS) potential in cells containing M-type mitochondria and partial switch to anaerobic energy production from glycolysis in the presence of egg cells^52^. Additionally, it has been shown that during the repetitive fusion and fission events between mitochondria the protein content inside them mixes faster than mtDNA nucleoids^53^. This was demonstrated in yeasts^54,55^ but if present in bivalves it partially could explain signal of Female-type mitochondrial proteins in bivalve male germ cells^9,56,57^. The fate of DUI species-derived mitogenomes and the proteins they produce appears to vary depending on the bivalve order or even family. This variability prevents us from making generalizations. If applicable to *Donax* spp., NADH proteins from F-type mitogenomes could potentially play an active role in complex I in matured sperm cells, even without the 'necessary' sequences within M-type mtDNA. Hypothetically the complex I function could also be replaced by another nuclear-encoded enzyme^47,58^ or unknown dehydrogenase subunits of NUMTs-like origin.

The phylogenetic framework based on mitogenomic F-type data was presented recently showing plethora of sequences from Donacidae family species that divided *Donax* genus into Easter Atlantic + Indo-Pacific and Western Atlantic + Eastern Pacific (both coasts of Americas) separate clades (separation in Late Cretaceous or mid Paleocene)^59^. It would be interesting to see if less related Donacidae M-type mitogenomes from around the globe (like *Donax californicus* Conrad, 1837 or *Latona dysoni* (Reeve, 1854) earlier *Donax*) will shed some light on the evolutionary fate of the mitochondrial complex I subunits and additional ORFs in this family.

In our phylogenetic reconstruction (Fig. 4) Male-type Tellinoidea mitogenomes group together on a phylogenetic tree (6 mitogenomes available). However, uncommon mtDNA features present in these mitogenomes, possibly associated with doubly uniparental inheritance, do not show a consistent pattern. In the *Donax* genus we see additional ORFs, lack of *nadh* subunits, and long extension at the 3’-end of *cox1* genes (Fig. 1). Additional ORFs and extensions of cytochrome c oxidase subunits have been speculated to function as a tagging mechanism allowing cells to distinguish M-type mitochondria from those of female-type^19,60–63^, but here we see extensions completely lacking homology to each other which seems to reduce the strength of this argument. The M-type mitogenomes of *Macoma balthica* and *Scrobicularia plana*, beside putative ORFs, contain extremely long open reading frame for *cox2* gene^43^ that recently was shown to have an intronic sequence^42^ but might also code for unusually long protein^64^. However, the *Gari togata* putative M-type mitogenome^41^ at the first glance shows no additional features, except for ORFs which may not necessarily be functional. Taking this into account, we assume that, similarly to freshwater mussels from Unionidae family^65^, DUI in Tellinoidea might have a singular point of origin. However, unlike in the case of unionids, the lack of consistency in type and sequence of specific features may suggest that none of them can function as a universal tag for M-type mitochondria or this tagging system (if exists) is much more complicated. Further research is needed to elucidate the functional significance of these peculiar M-type mitogenomes.

## Methods

The samples of *Donax trunculus* were collected from natural beds in Vilarrube (northwestern Spain), while *Donax vittatus* and *Donax semistriatus* specimens were gathered around Portuguese coast (Table 1). Clams were transported to laboratory, sectioned, and stored refrigerated in 70% ethanol until further use. From each species one male individual was identified by gonad analysis under the light microscope (presence of sperm cells and lack of egg cells). Next total DNA extraction from gonads (some contamination with somatic cells was possible) was performed according to modified CTAB method^19,66^. Isolated DNA was then quality checked by 1% agarose gel electrophoresis, and Epoch microplate spectrophotometer. For NGS sequencing, (NovaSeq platform, TruSeq NGS library 2 × 150 bp) samples were send to Macrogen (Korea). Mitogenomes were assembled with NOVOplasty^31^^.^. In each case the assembler reported the mitogenome as circularized, and the verification by mapping the raw reads back on the assembly in CLC Genomics Workbench (QIAGEN) confirmed the integrity of the assemblies. For mitogenomes annotation and visualisation of circular diagrams MITOCONSTRICTOR scripts (available from GitHub) with default parameters combining outputs from several bioinformatic tools were used (Wise2^33^, TMHMM^46^, Infernal^35^, ARWEN^34^, GLIMMER^67^, CRITICA^68^, BLAST + ^32^, Vmatch^69^, ViennaRNA Package^70^, EMBOSS^71^). Mitogenome sequences can be found in GenBank under OR416182, OR416183, OR416184 accession numbers. Additional analyses of mitogenomic features were performed in MEGA7^36^ (alignments and pairwise distances), DnaSP^72^ (sliding window analysis), and AlphaFold2^73^ (models of 3D protein structures, available in supplementary data).

**Table 1:** *Donax* spp. sampling details.

Species	Location	Country	Latitude	Longitude
*D. semistriatus*	Monte Gordo	Portugal	37.167	− 7.503
*D. trunculus*	Vilarrube	Spain	43.644	− 8.077
*D. vittatus*	Mira-Vagueira	Portugal	40.614	− 8.769

Phylogenetic reconstruction was done by comparison of results from two programs Beast2^39^ (Bayesian phylogenetic analysis) and Iqtree2^40^ (Maximum likelihood). For both programs, 7 partitions were used containing alignments of 7 genes (*cox1-cox3, cytb, atp6,* 12S rRNA, 16S rRNA) made in MEGA7 software^36^, Gap opening and extension penalty set to 3. All *nadh,* and *atp8* genes were excluded from analysis due to the missing annotations for them in a few of the mitogenomes. Appropriate substitution models were assigned to partitions based on implemented in the programs model test tools. Based on the data available in GenBank database (September 2022) 23 mitogenomes from superfamily Tellinoidea were chosen. The Beast2 analysis was conducted in 4 replicates for 10^7^ generations, with sampling at every 10,000^th^ step. The analysis utilized a relaxed log-normal clock, GTR + I + 4G substitution model, and Yule prior for the common tree. Afterwards, the effective sample size for estimated parameters > 200 was checked in Tracer^74^. The Iqtree2 ran with an ultrafast bootstrap approximation parameter set to 100,000 replicates. The TVM + I + G4 substitution model was assigned to 12S and 16S rRNA, the GTR + I + G4 to *cox1* and *cytb*, and the TVM + G4 to *cox2*, *cox3*, *atp6*.

### Supplementary Information

Supplementary Information.

## Data Availability

The mitogenomic sequences and annotations generated and analysed during the current study are available in the GenBank database, under OR416182, OR416183, OR416184 accession numbers, as well as in Supplementary data.
