# Supplementary material for: The absence of canonical respiratory complex I subunits in male-type mitogenomes of three Donax species
Source: Sci Rep. 2024 Jun 24;14:14465. doi: 10.1038/s41598-024-63764-8 (PMC11196677; doi:10.1038/s41598-024-63764-8)
Supplement: Supplementary file 1 — Supplementary Information. [file 41598_2024_63764_MOESM1_ESM.pdf]

Supplementary data for:

The absence of canonical respiratory complex I subunits in male-type mitogenomes of three *Donax* species.

Artur Burzyński, Beata Śmietanka, Jenyfer Fernández-Pérez, Marek Lubośny

Table 1 M-type *Donax vittatus* mtDNA annotation table (OR416182)

Name	Type	Start	Stop	Size
tRNA-Phe	tRNA	568	630	63
ORF1	CDS	822	4535	3714
Cox2-3'	CDS	4853	5278	426
tRNA-Val	tRNA	5296	5359	64
tRNA-Met	tRNA	5452	5514	63
Atp8	CDS	5527	5676	150
5'-*Cox2*	CDS	5698	6711	1014
tRNA-Ser	tRNA	6871	6939	69
tRNA-Trp	tRNA	6941	7000	60
ORF2	CDS	7005	7919	915
tRNA-Gly	tRNA	7945	8006	62
ORF3	CDS	8007	8804	798
12S rRNA	rRNA	9101	9798	698
5'-16S rRNA	rRNA	9915	10298	384
16S rRNA-3'	rRNA	10915	11715	801
Atp6	CDS	11716	12411	696
Cox3	CDS	12412	13338	927
tRNA-Pro	tRNA	13339	13400	62
tRNA-Gln	tRNA	13401	13465	65
tRNA-Cys	tRNA	13465	13527	63
tRNA-Ala	tRNA	13526	13588	63
Cox1	CDS	13618	16224	2607
tRNA-His	tRNA	16514	16577	64
tRNA-Ser	tRNA	16576	16642	67
tRNA-Glu	tRNA	16641	16704	64
tRNA-Ile	tRNA	16704	16769	66
tRNA-Lys	tRNA	16770	16832	63
tRNA-Tyr	tRNA	16834	16897	64
tRNA-Thr	tRNA	16896	16958	63
tRNA-Leu	tRNA	16966	17025	60
tRNA-Asp	tRNA	17028	17087	60
tRNA-Leu	tRNA	17088	17150	63
tRNA-Asn	tRNA	17148	17211	64
tRNA-Arg	tRNA	17217	17274	58
Cytb	CDS	17276	18583	1308

Table 2 M-type *Donax trunculus* mtDNA annotation table (OR416183)

Name	Type	Start	Stop	Size
tRNA-Phe	tRNA	474	537	64
ORF1	CDS	540	4685	4146
Cox2-3’	CDS	5199	5666	468
tRNA-Val	tRNA	6367	6429	63
tRNA-Met	tRNA	6428	6491	64
Atp8	CDS	6503	6649	147
5’-*Cox2*	CDS	6678	7554	877
tRNA-Ser	tRNA	7554	7620	67
tRNA-Trp	tRNA	7620	7683	64
tRNA-Gly	tRNA	7684	7746	63
ORF2	CDS	7750	8745	996
ORF3	CDS	8779	9750	972
12S rRNA	rRNA	10141	10843	703
5'-16S rRNA	rRNA	10962	11358	397
16S rRNA-3'	rRNA	11413	12202	790
Atp6	CDS	12398	13093	696
Cox3	CDS	13094	14006	913
tRNA-Pro	tRNA	14006	14067	62
tRNA-Gln	tRNA	14069	14134	66
tRNA-Cys	tRNA	14133	14195	63
tRNA-Ala	tRNA	14195	14258	64
Cox1	CDS	14259	17006	2748
tRNA-His	tRNA	17046	17104	59
tRNA-Ser	tRNA	17106	17167	62
tRNA-Ile	tRNA	17231	17298	68
tRNA-Glu	tRNA	17402	17465	64
tRNA-Lys	tRNA	17529	17592	64
tRNA-Tyr	tRNA	17734	17797	64
tRNA-Thr	tRNA	17796	17855	60
tRNA-Leu	tRNA	17856	17915	60
tRNA-Asp	tRNA	17915	17974	60
tRNA-Leu	tRNA	17981	18043	63
tRNA-Asn	tRNA	18043	18104	62
tRNA-Arg	tRNA	18103	18163	61
Cytb	CDS	18164	19507	1344

Table 3 M-type *Donax semiestriatus* mtDNA annotation table (OR416184)

Name	Type	Start	Stop	Size
tRNA-Phe	tRNA	568	631	64
ORF1	CDS	697	4371	3675
Cox2-3'	CDS	4494	4943	450
tRNA-Val	tRNA	4957	5018	62
tRNA-Met	tRNA	5017	5078	62
Atp8	CDS	5091	5240	150
5'-*Cox2*	CDS	5375	6418	1044
tRNA-Ser	tRNA	6422	6490	69
tRNA-Trp	tRNA	6491	6552	62
ORF2	CDS	6551	7471	921
tRNA-Gly	tRNA	7508	7569	62
ORF3	CDS	7570	8370	801
12S rRNA	rRNA	8642	9336	695
5'-16S rRNA	rRNA	9453	9836	384
16S rRNA-5'	rRNA	10217	10982	766
Atp6	CDS	10985	11680	696
Cox3	CDS	11681	12608	928
tRNA-Pro	tRNA	12610	12670	61
tRNA-Gln	tRNA	12671	12734	64
tRNA-Cys	tRNA	12733	12793	61
tRNA-Ala	tRNA	12793	12854	62
Cox1	CDS	12854	15244	2391
tRNA-His	tRNA	15485	15545	61
tRNA-Ser	tRNA	15545	15611	67
tRNA-Glu	tRNA	15610	15673	64
tRNA-Ile	tRNA	15674	15741	68
tRNA-Lys	tRNA	15742	15804	63
tRNA-Tyr	tRNA	15805	15868	64
tRNA-Thr	tRNA	15867	15929	63
tRNA-Leu	tRNA	15941	16001	61
tRNA-Asp	tRNA	16002	16061	60
tRNA-Leu	tRNA	16062	16123	62
tRNA-Asn	tRNA	16121	16185	65
tRNA-Arg	tRNA	16185	16246	62
Cytb	CDS	16246	17517	1272

Table 4 Coverage of *Donax* spp. M-type mtDNA genes with sequencing reads

Gene coverage	*D. trunculus*	*D. semistriatus*	*D. vittatus*
ORF1	288.31	490.05	133.51
Cox2-3'	257.88	434.19	132.47
Atp8	252.16	382.97	113.73
5'-Cox2	278.63	462.09	127.68
ORF2	260.31	465.04	134.26
ORF3	270.19	470.31	142.08
12S	219.88	265.03	102.49
5'-16S<br>16S-3'	251.82	297.28	115.60
Atp6	286.99	471.00	146.43
Cox3	311.81	382.23	123.50
Cox1	290.80	433.75	138.12
CytB	243.73	317.03	94.56

Table 5 Coverage of *D. trunculus* mitogenomes with sequencing reads (144 621 144 reads)

mitogenome	mapping on:	no. of reads	avg. coverage
M mtDNA	OR416183	35812	268.88
F mtDNA	KY780364	1083	10.53

Table 6 Coverage of *D. semistriatus* mitogenomes with sequencing reads (159 866 686 reads)

mitogenome	mapping on:	no. of reads	avg. coverage
M mtDNA	OR416184	49320	422.11
F mtDNA	KY780363	178	1.66

Table 7 Coverage of *D. vittatus* mitogenomes with sequencing reads (151 532 424 reads)

mitogenome	mapping on:	no. of reads	avg. coverage
M mtDNA	OR416182	17403	132.21
F mtDNA	KY780366	66	0.58

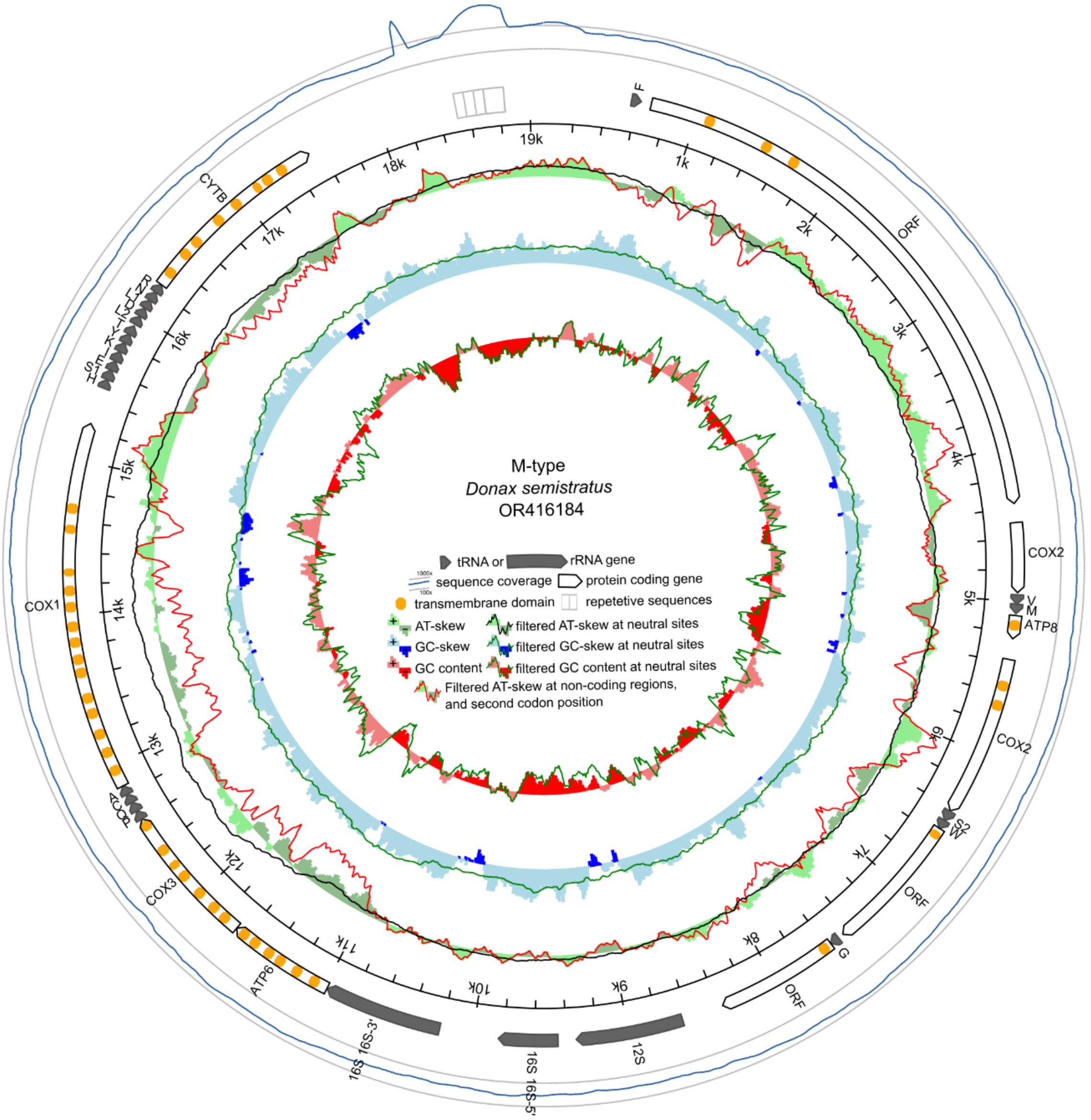

Figure 1 Genetic map of the M-type *Donax semistratus* mitogenome

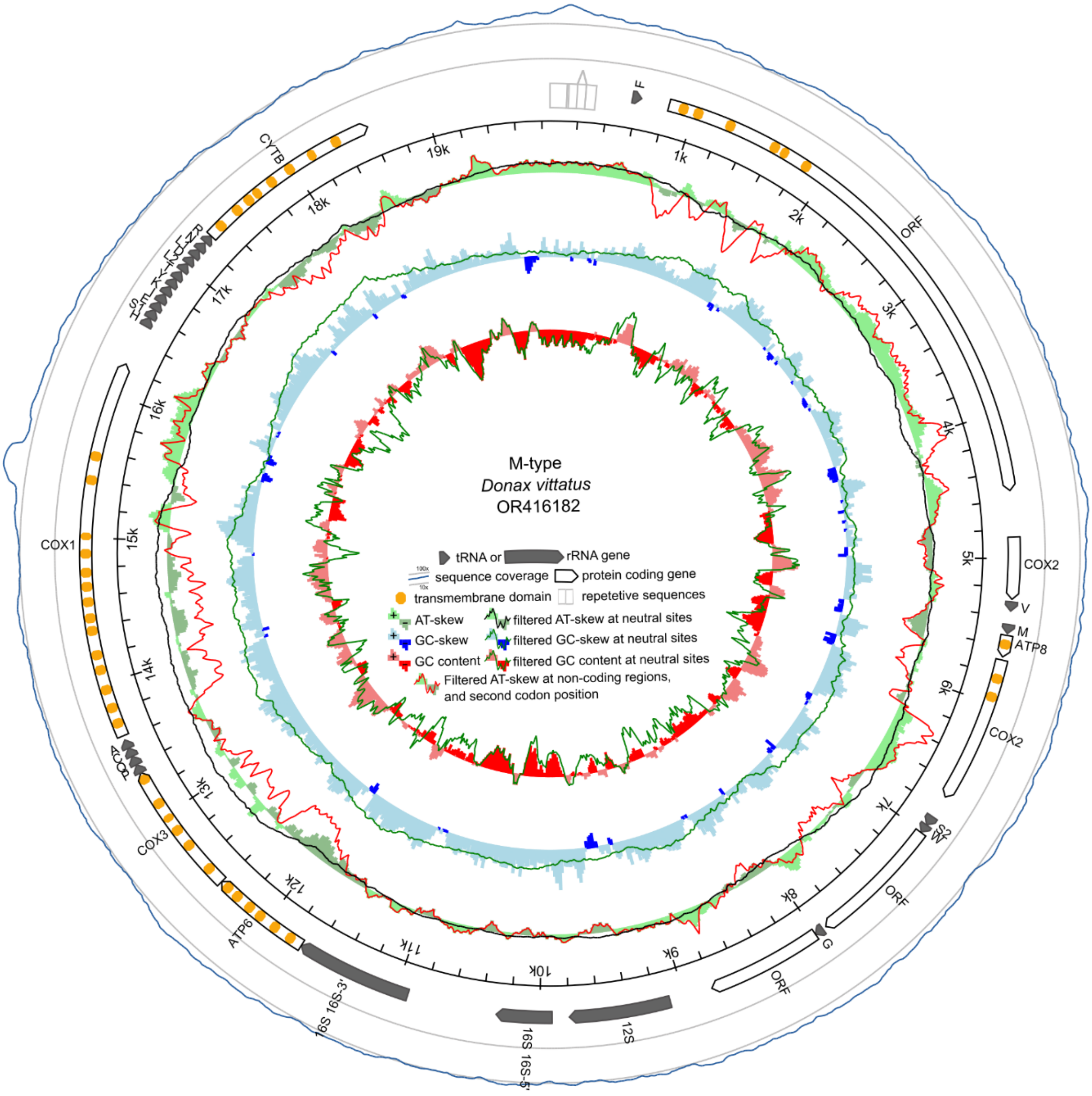

Figure 2 Genetic map of the M-type *Donax vittatus* mitogenome

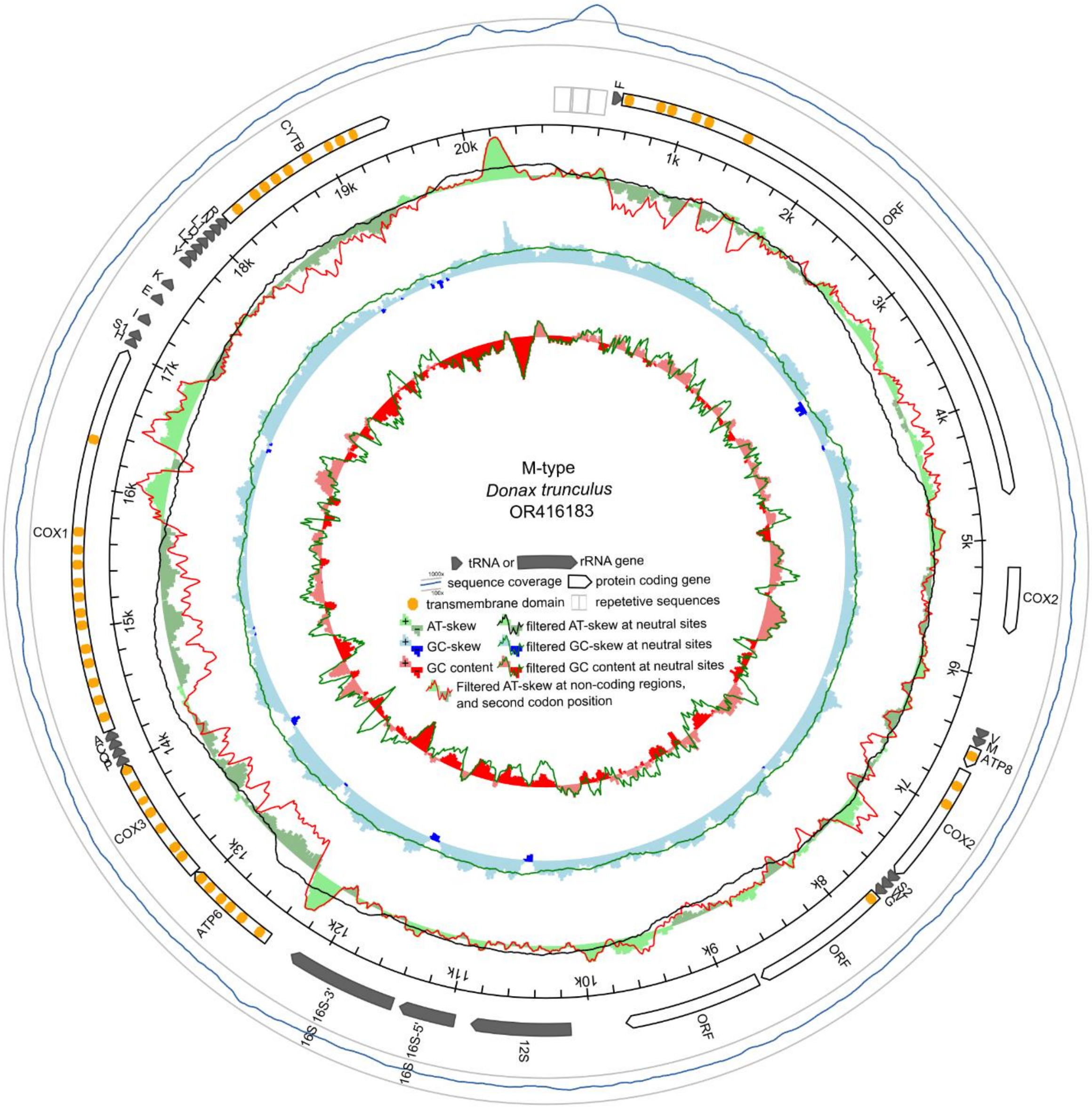

Figure 3 Genetic map of the M-type *Donax trunculus* mitogenome

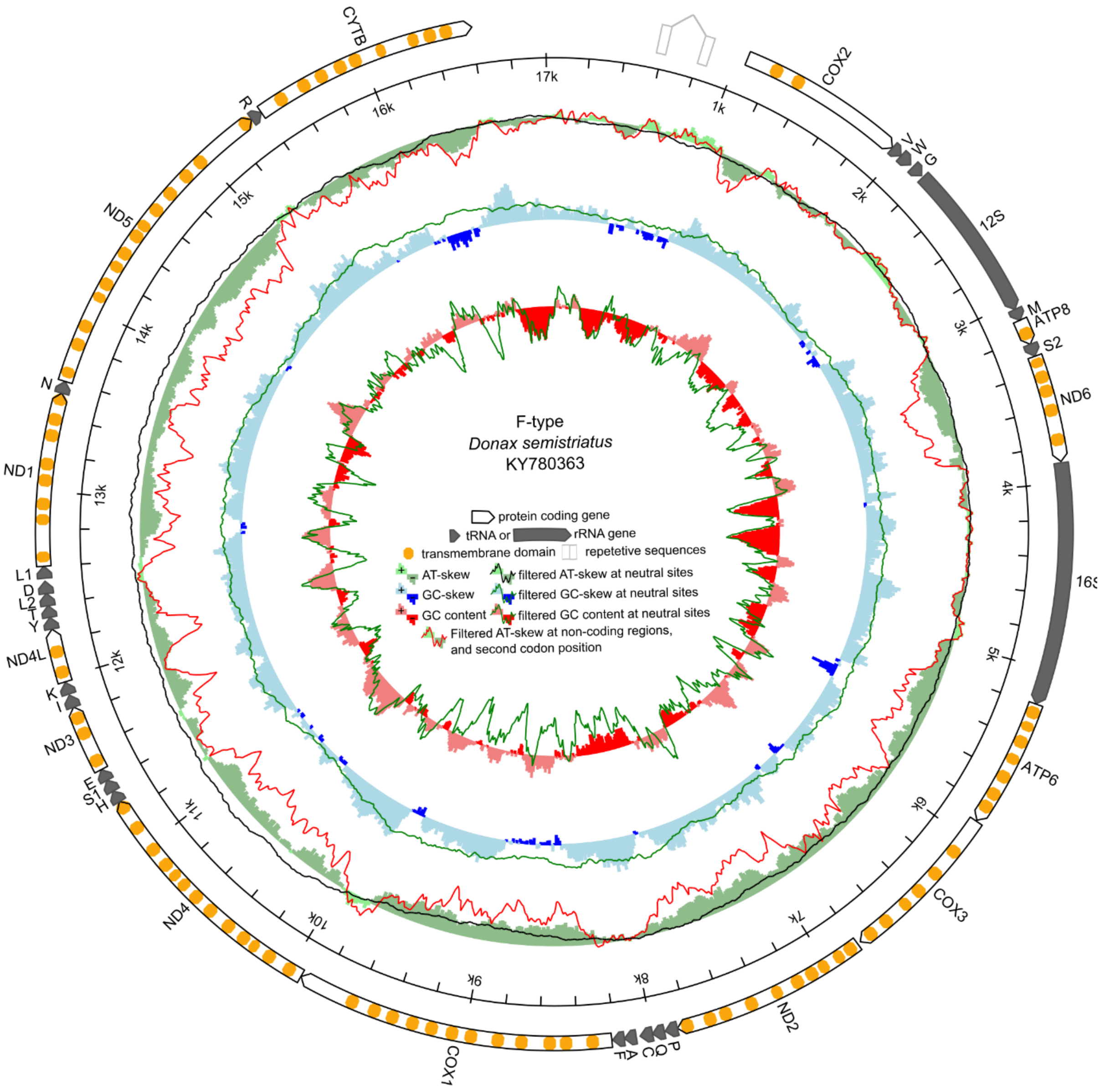

Figure 4 Example of genetic map of the F-type *Donax semistratus* mitogenome

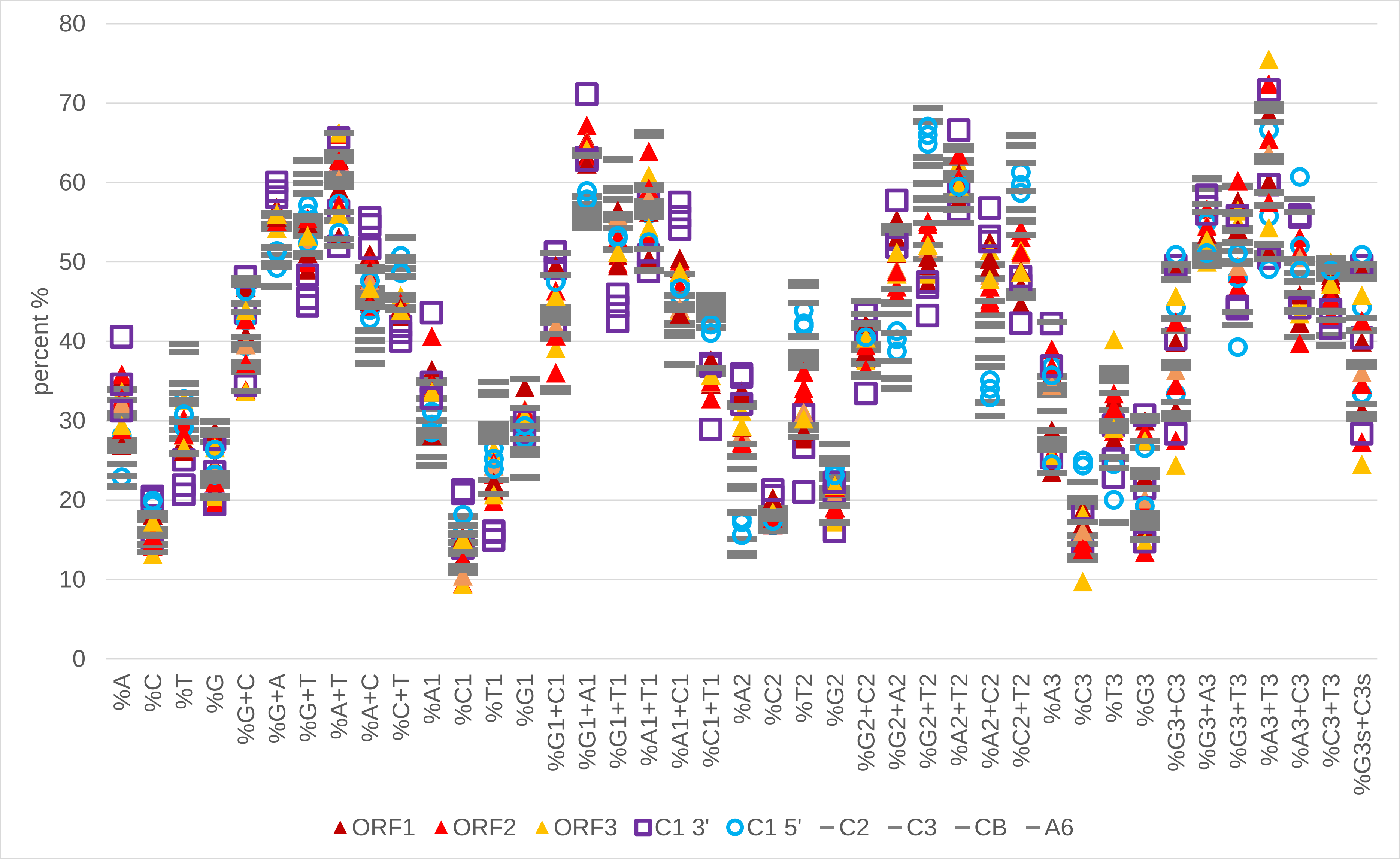

Figure 5 Nucleotide composition of M-type *Donax* mitochondrial genes

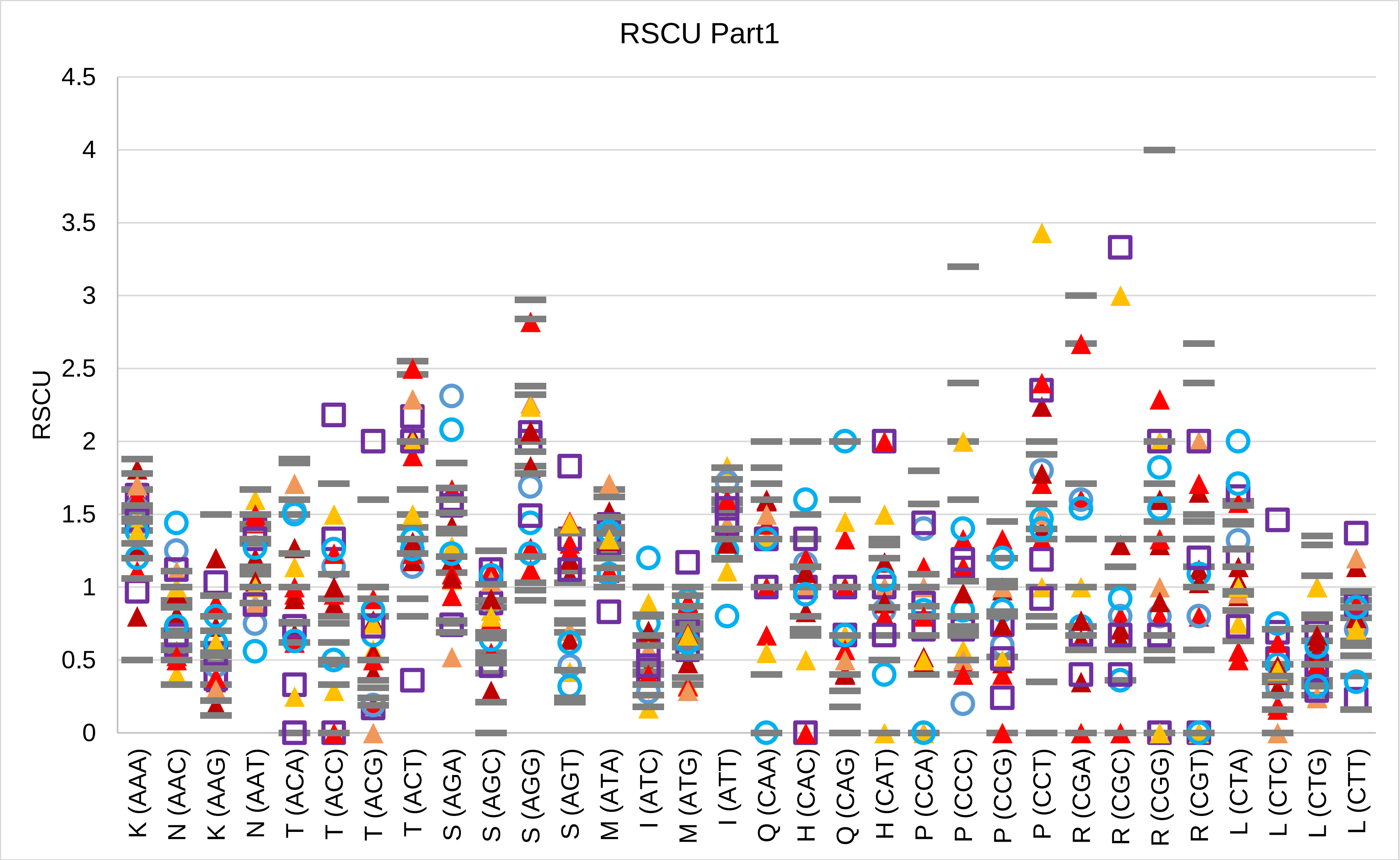

Figure 6 RSCU for three M-type *Donax* mitochondrial protein coding genes (Part 1)

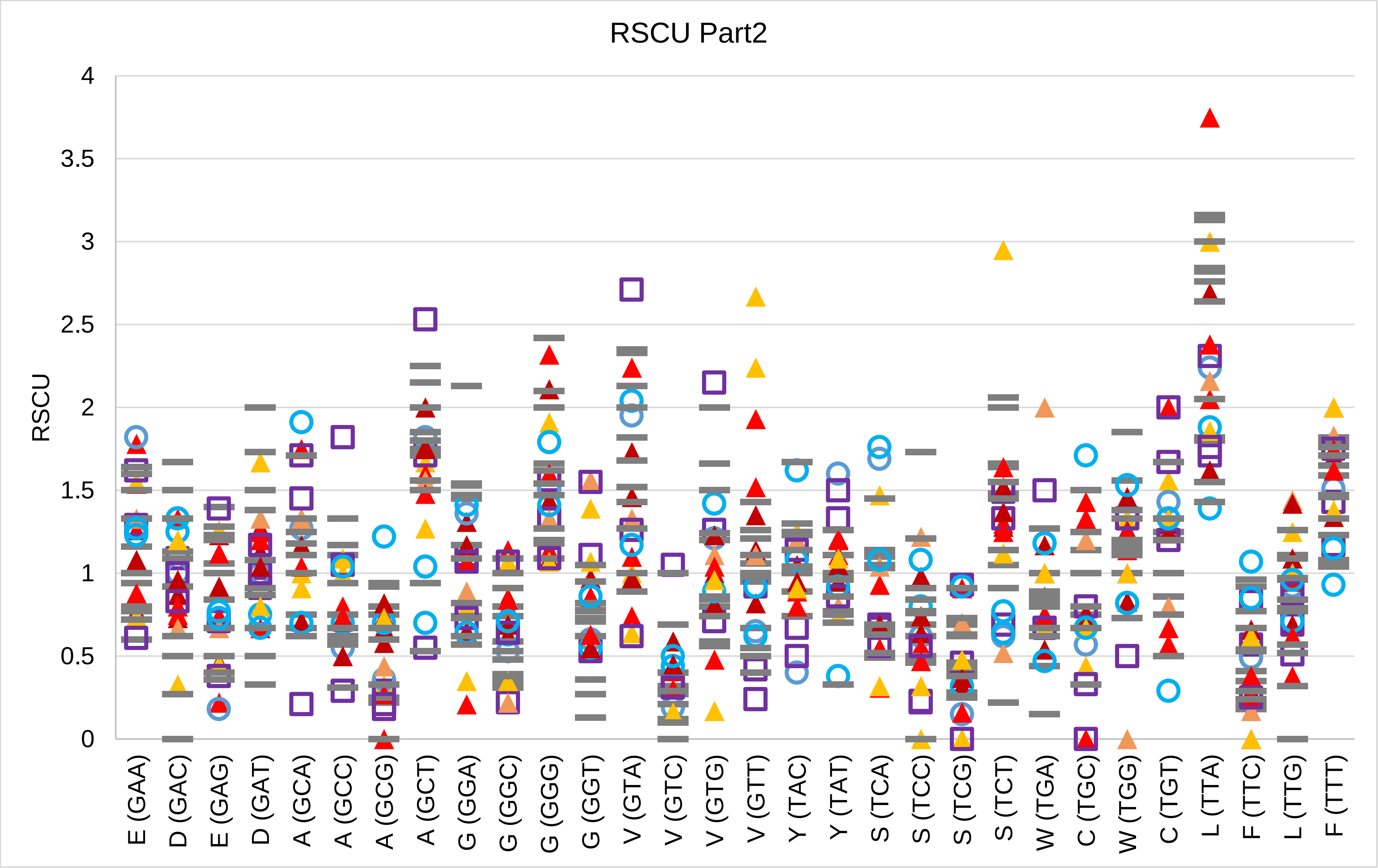

Figure 7 RSCU for three M-type *Donax* mitochondrial protein coding genes (Part 2)

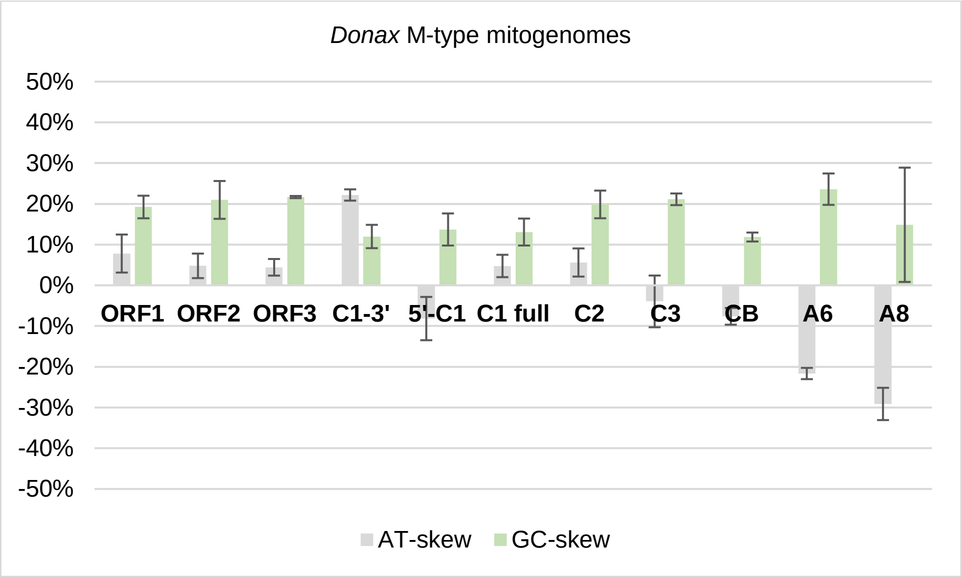

Figure 8 Average AT-skew and GC-skew for genes from three M-type *Donax* mitogenomes

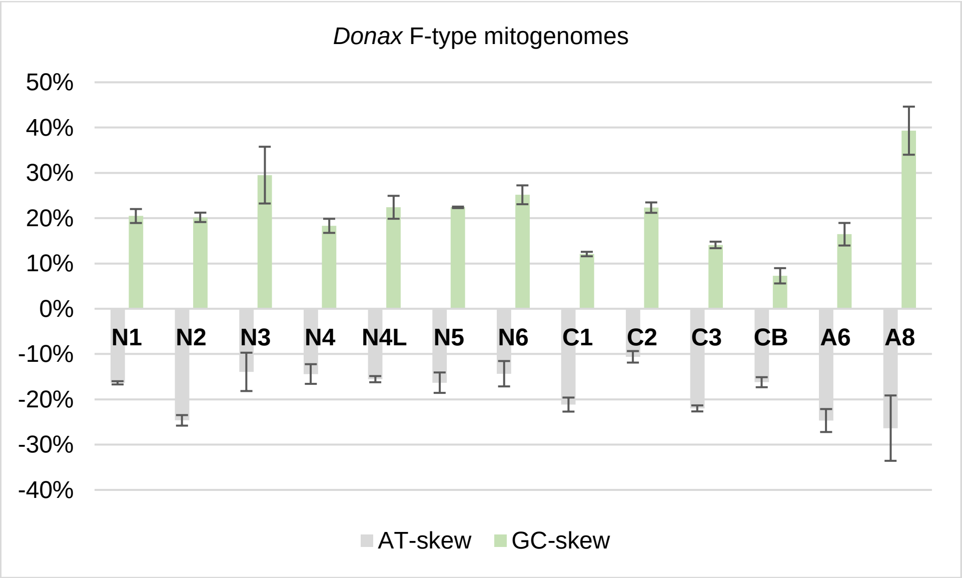

Figure 9 Average AT-skew and GC-skew for genes from three F-type *Donax* mitogenomes

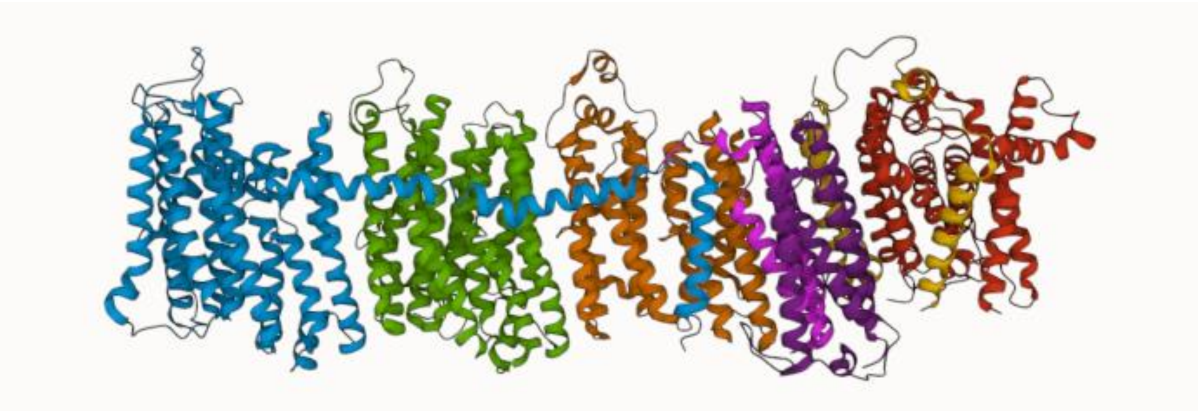

Figure 10 Visualisation of mitochondrial-encoded subunits of complex I (extracted from 7VBL PDB structure). Red indicated subunit NADH1; orange NADH2; yellow NADH3; green NADH4; pink NADH4L; blue NADH5; violet NADH6.

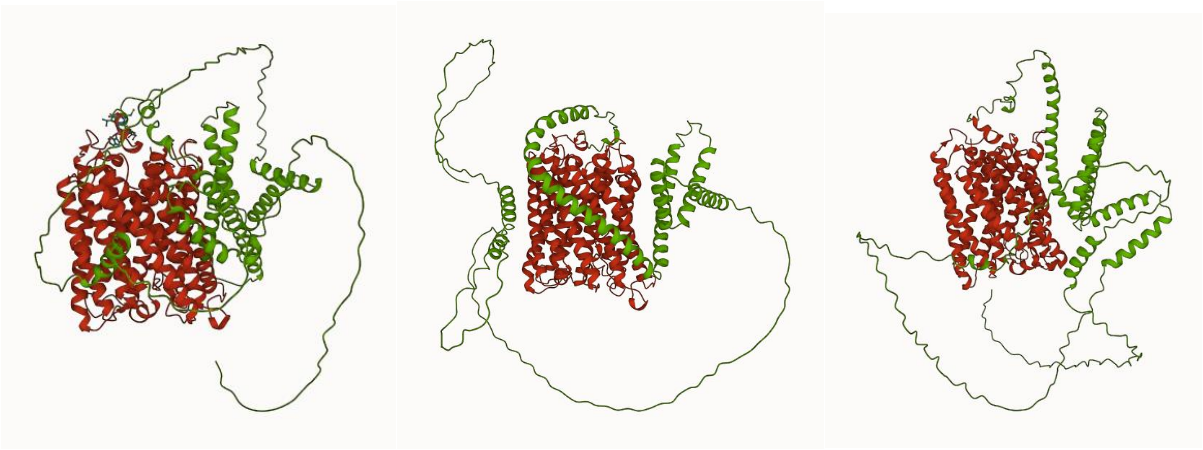

Figure 11 AlphaFold2 prediction of COX1 with extension *Donax vittatus* (three different structures). Red indicates conserved 5’ COX1 protein region; green indicated 3’ COX1 extension.

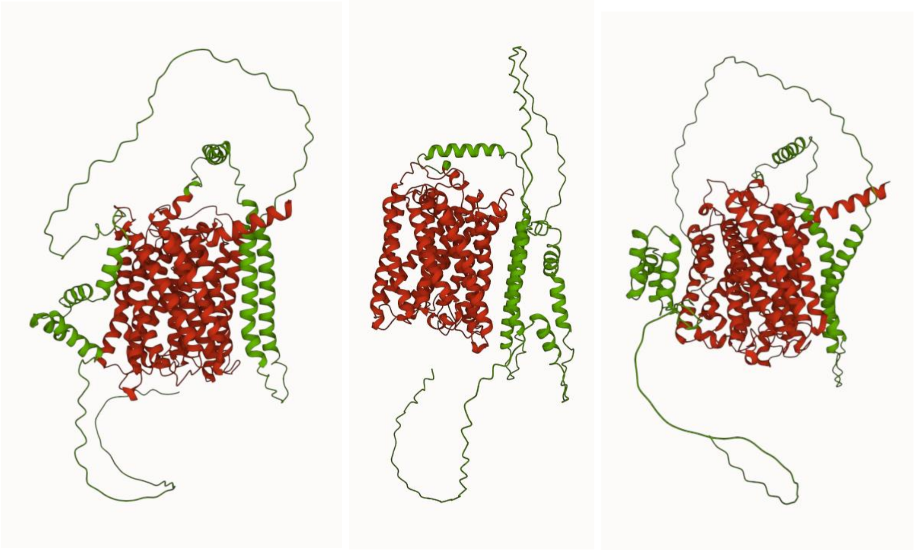

Figure 12 AlphaFold2 prediction of COX1 with extension *Donax semiestriatus* (three different structures). Red indicates conserved 5’ COX1 protein region; green indicated 3’ COX1 extension.

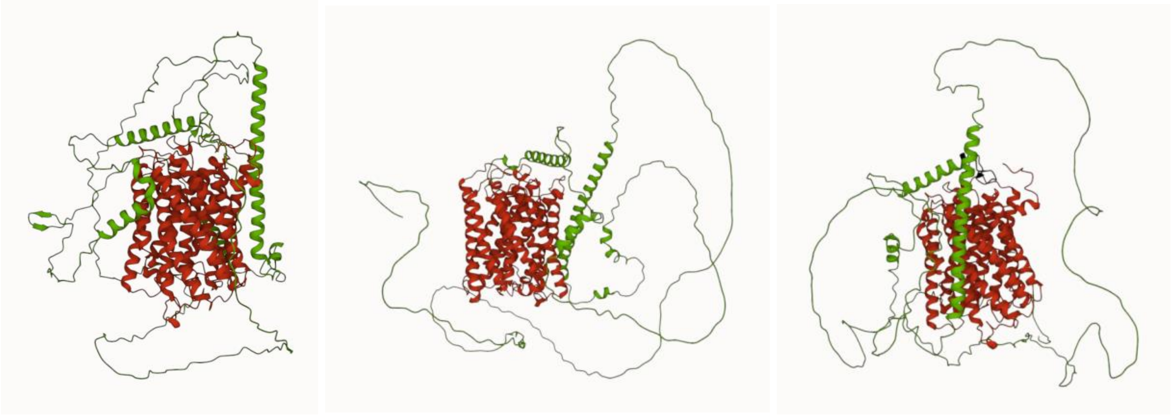

Figure 13 AlphaFold2 prediction of COX1 with extension *Donax trunculus* (three different structures). Red indicates conserved 5’ COX1 protein region; green indicated 3’ COX1 extension.

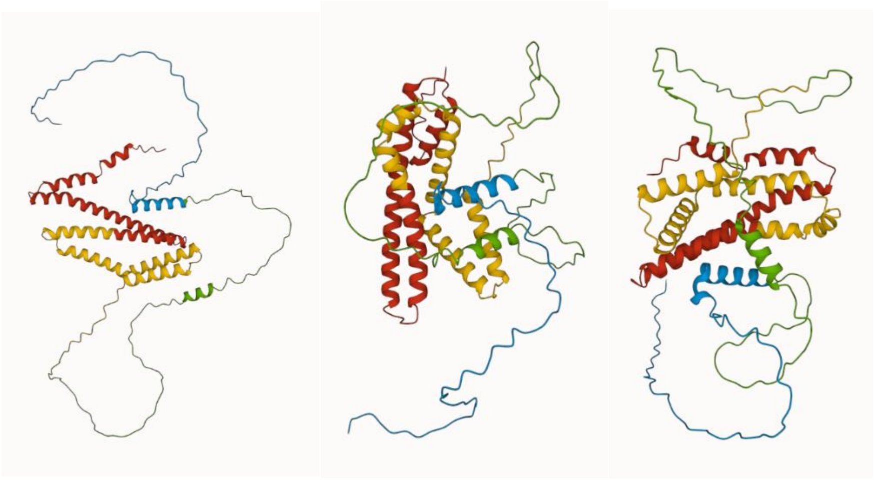

Figure 14 AlphaFold2 *Donax vittatus* prediction of COX1 extension (only extension). Red indicates first 100 amino acids; yellow 101-200 aa; green 201-300 aa; blue 301-400+ aa.

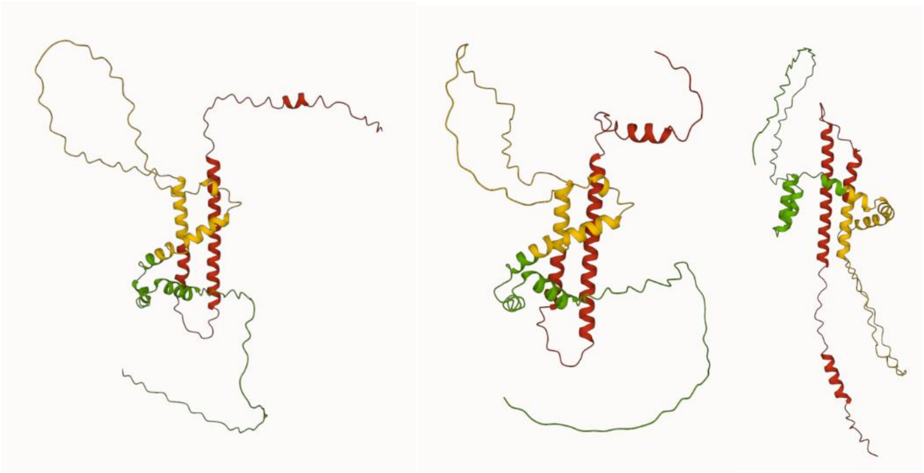

Figure 15 AlphaFold2 *Donax semiestriatus* prediction of COX1 extension (only extension). Red indicates first 100 amino acids; yellow 101-200 aa; green 201-300 aa;

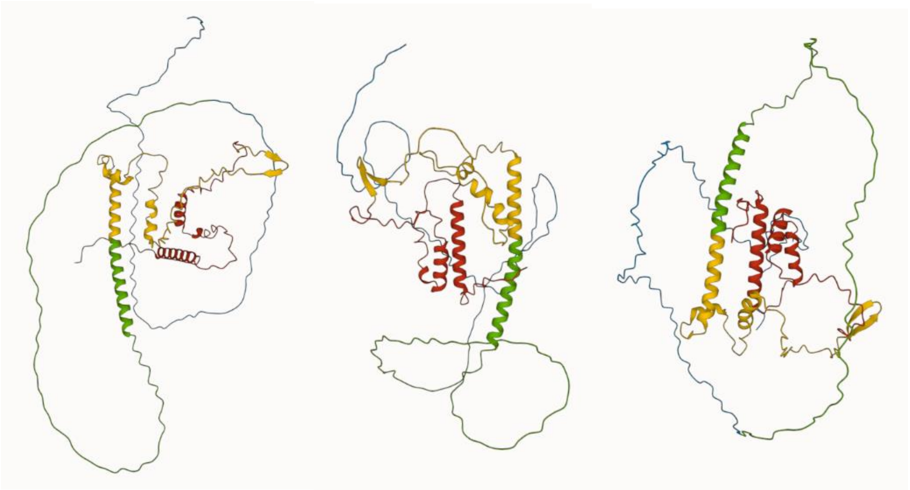

Figure 16 AlphaFold2 *Donax trunculus* prediction of COX1 extension (only extension). Red indicates first 100 amino acids; yellow 101-200 aa; green 201-300 aa; blue 301-400+ aa.

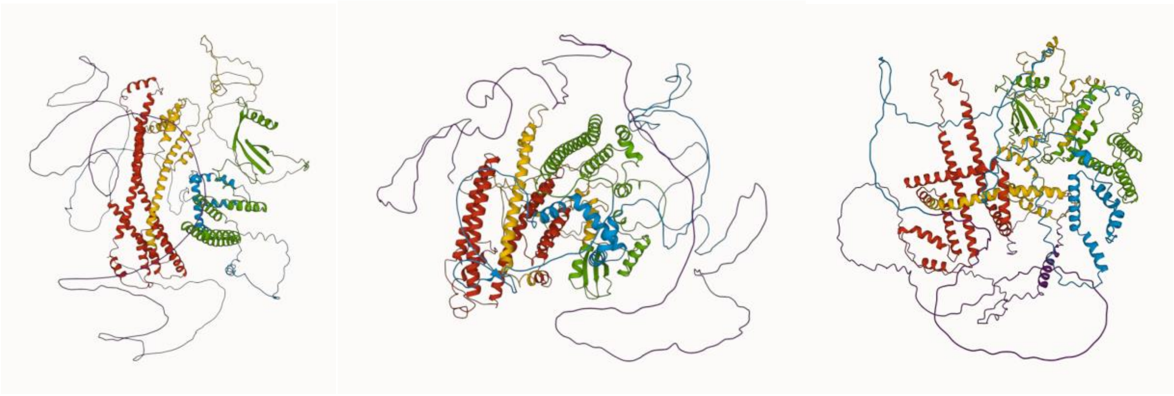

Figure 17 AlphaFold2 *Donax vittatus* prediction of ORF1 (three different structures). Red indicates first 250 amino acids; yellow 251-500 aa; green 501-750 aa; blue 751-1000 aa; violet 1001-1250 aa.

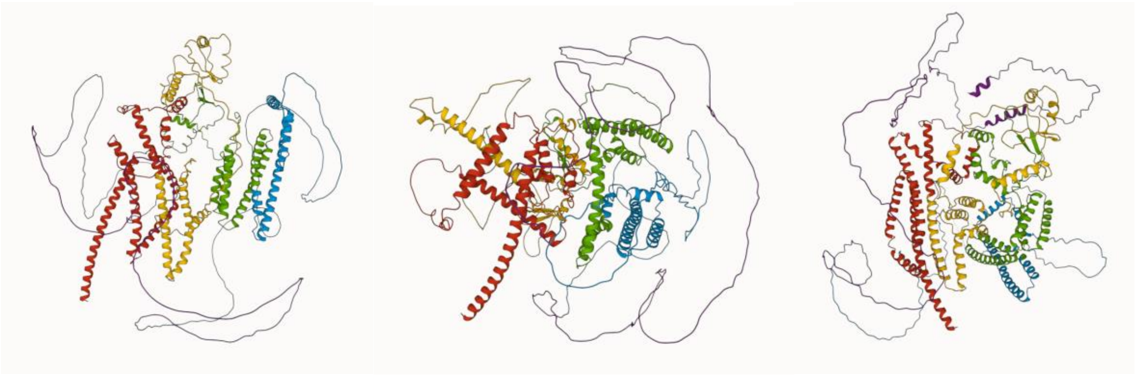

Figure 18 AlphaFold2 *Donax semistriatus* prediction of ORF1 (three different structures). Red indicates first 250 amino acids; yellow 251-500 aa; green 501-750 aa; blue 751-1000 aa; violet 1001-1250 aa.

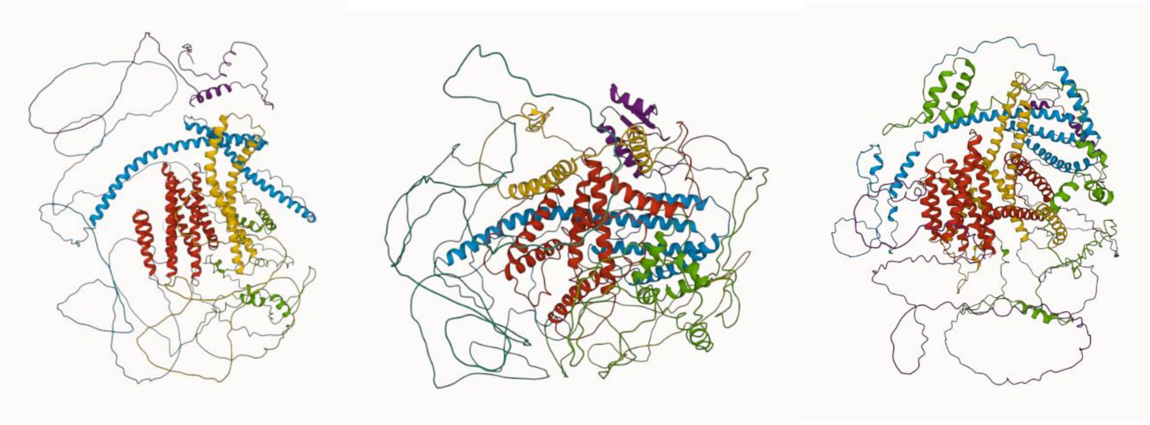

Figure 19 AlphaFold2 *Donax trunculus* prediction of ORF1 (three different structures). Red indicates first 250 amino acids; yellow 251-500 aa; green 501-750 aa; blue 751-1000 aa; violet 1001-1250+ aa.

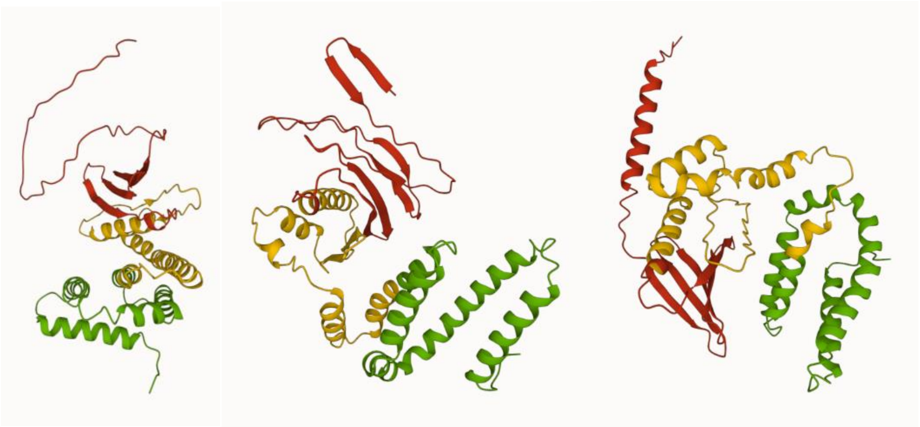

Figure 20 AlphaFold2 *Donax vittatus* prediction of ORF2 (three different structures). Red indicates first 100 amino acids; yellow 101-200 aa; green 201-300+ aa;

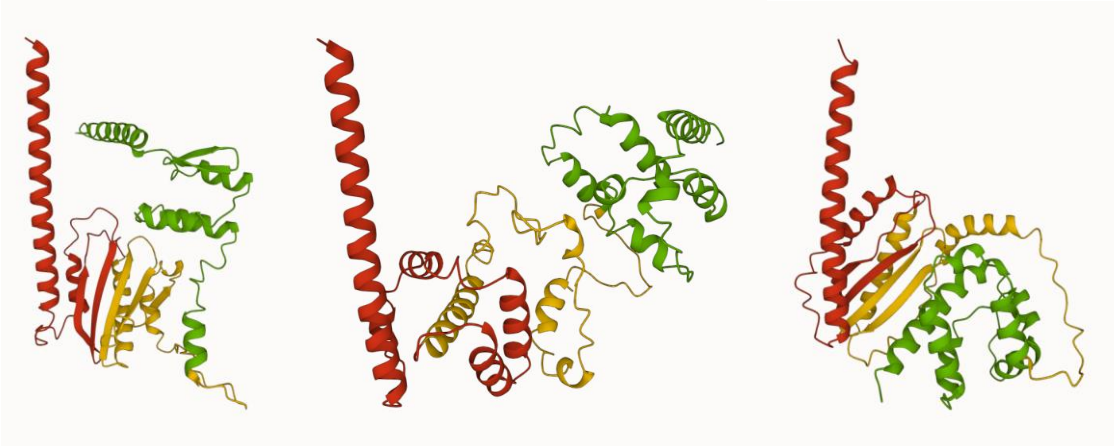

Figure 21 AlphaFold2 *Donax semistriatus* prediction of ORF2 (three different structures). Red indicates first 100 amino acids; yellow 101-200 aa; green 201-300+ aa;

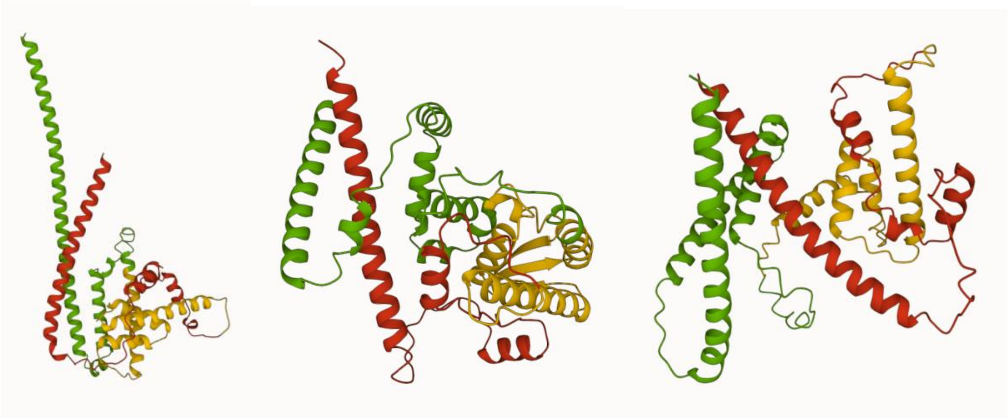

Figure 22 AlphaFold2 *Donax trunculus* prediction of ORF2 (three different structures). Red indicates first 100 amino acids; yellow 101-200 aa; green 201-300+ aa;

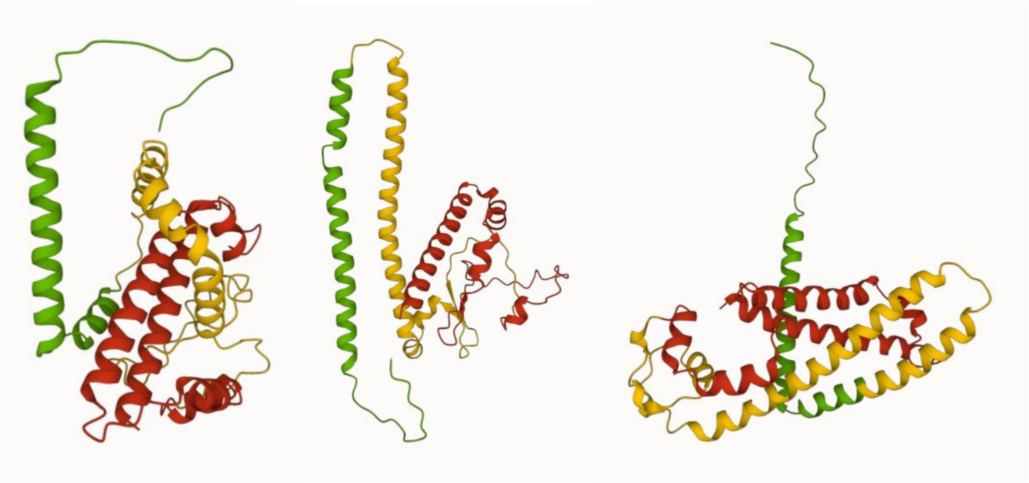

Figure 23 AlphaFold2 *Donax vittatus* prediction of ORF3 (three different structures). Red indicates first 100 amino acids; yellow 101-200 aa; green 201-300+ aa;

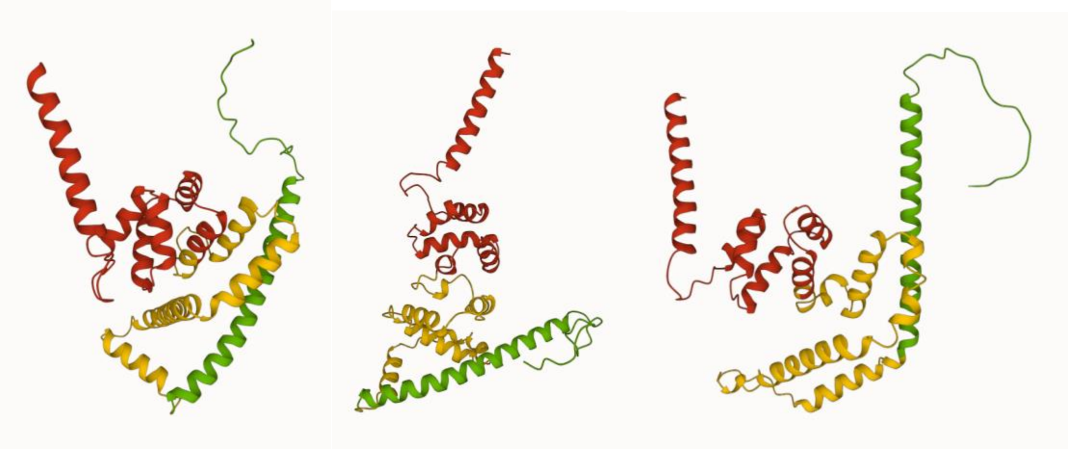

Figure 24 AlphaFold2 *Donax semistriatus* prediction of ORF3 (three different structures). Red indicates first 100 amino acids; yellow 101-200 aa; green 201-300+ aa;

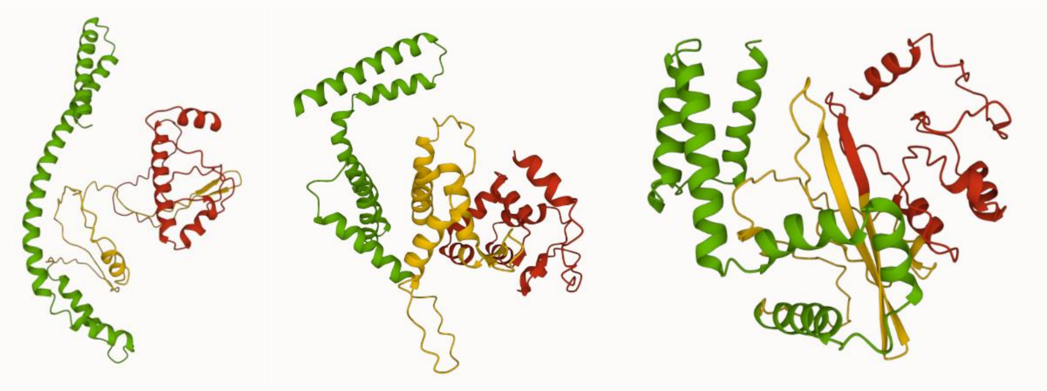

Figure 25 AlphaFold2 *Donax trunculus* prediction of ORF3 (three different structures). Red indicates first 100 amino acids; yellow 101-200 aa; green 201-266 aa;

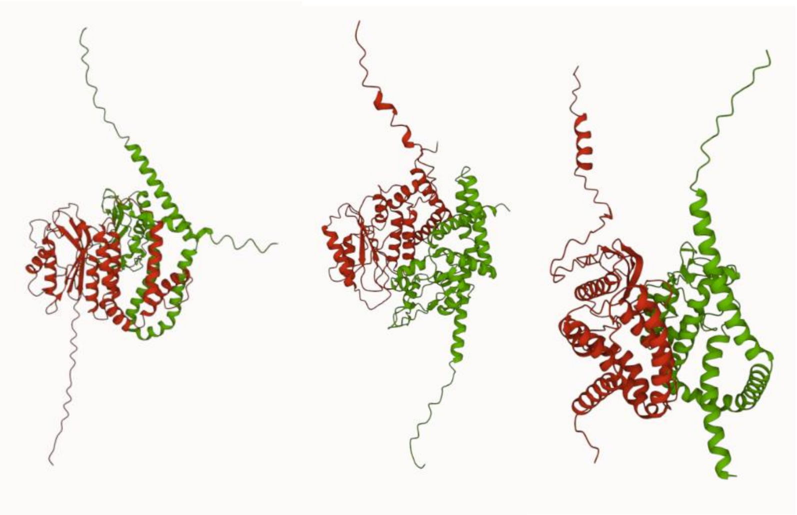

Figure 26 AlphaFold2 *Donax vittatus* prediction of ORF2:ORF3 complex (three different structures). Red indicates ORF2 and green ORF3.

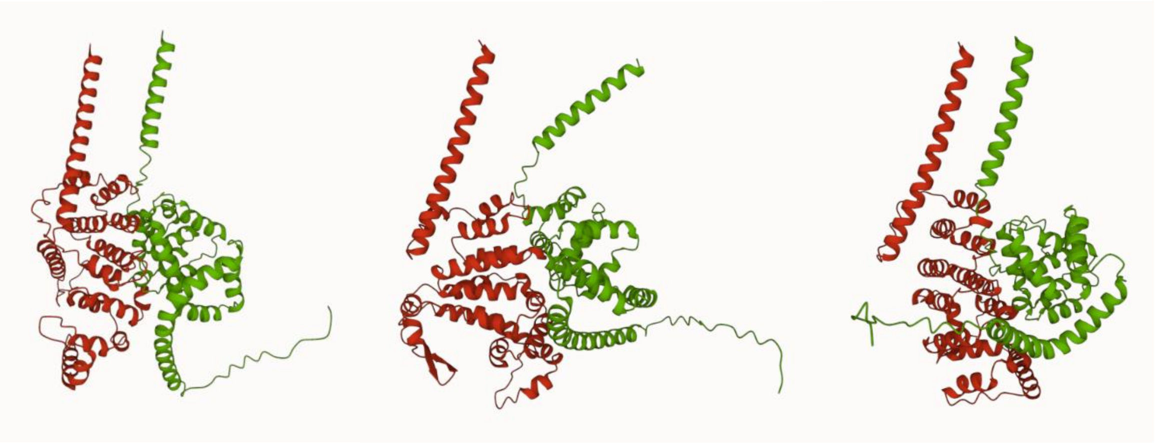

Figure 27 AlphaFold2 *Donax semistriatus* prediction of ORF2:ORF3 complex (three different structures). Red indicates ORF2 and green ORF3.

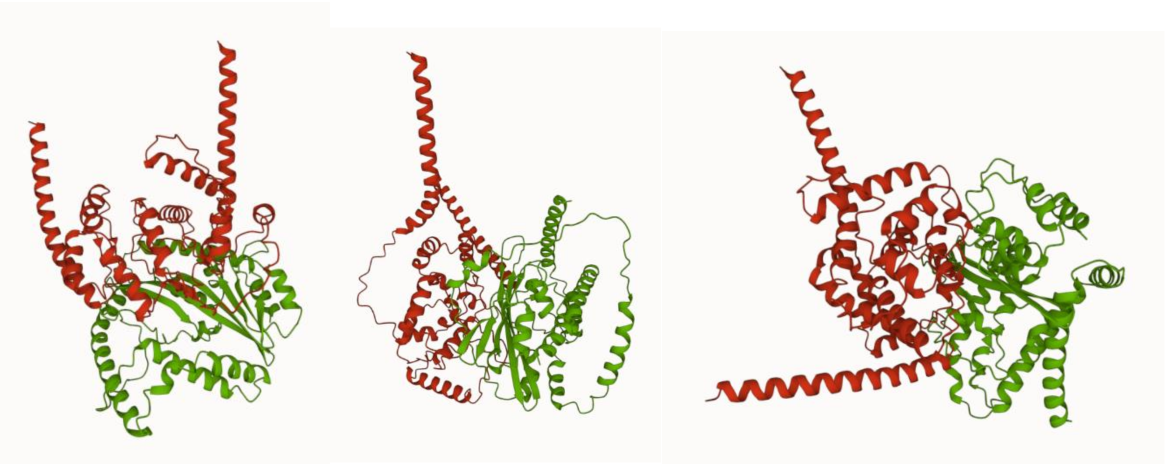

Figure 28 AlphaFold2 *Donax trunculus* prediction of ORF2:ORF3 complex (three different structures). Red indicates ORF2 and green ORF3.

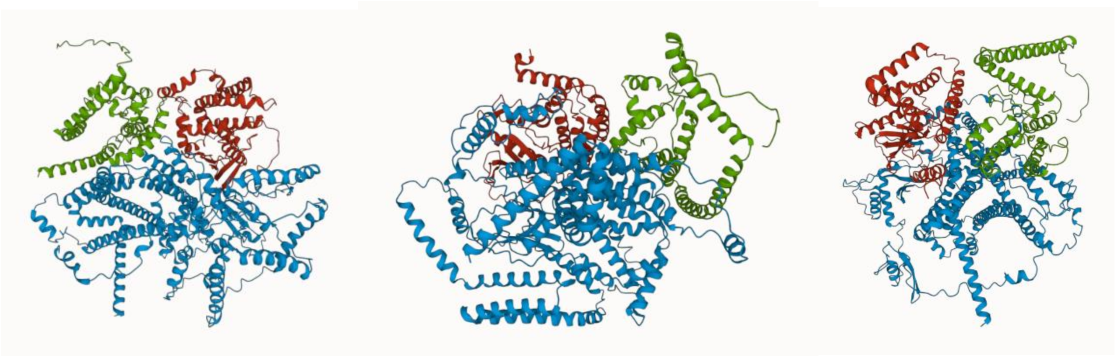

Figure 29 AlphaFold2 *Donax vittatus* prediction of ORF1:ORF2:ORF3 complex (three different structures). Blue indicates fragment of ORF1; red ORF2 and green ORF3 (only fragment of ORF1 due to the limitation of the modeling software 1400 amino acids in total)

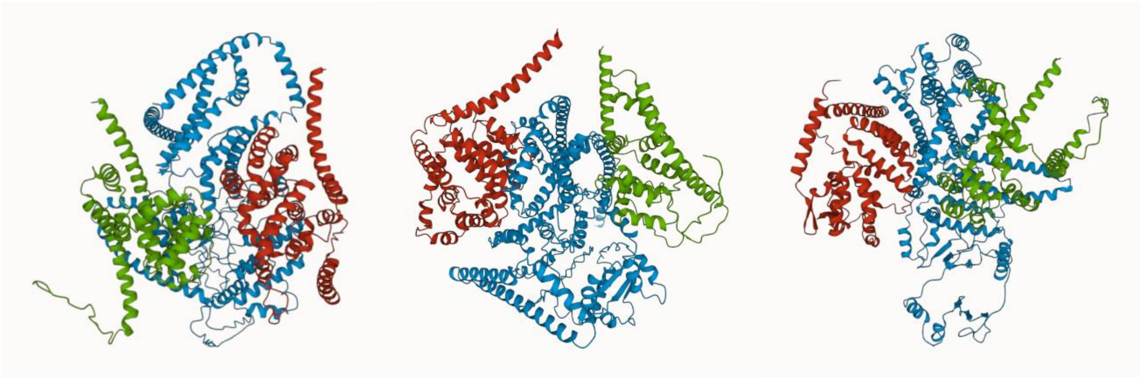

Figure 30 AlphaFold2 *Donax semistriatus* prediction of ORF1:ORF2:ORF3 complex (three different structures). Blue indicates fragment of ORF1; red ORF2 and green ORF3 (only fragment of ORF1 due to the limitation of the modeling software 1400 amino acids in total)

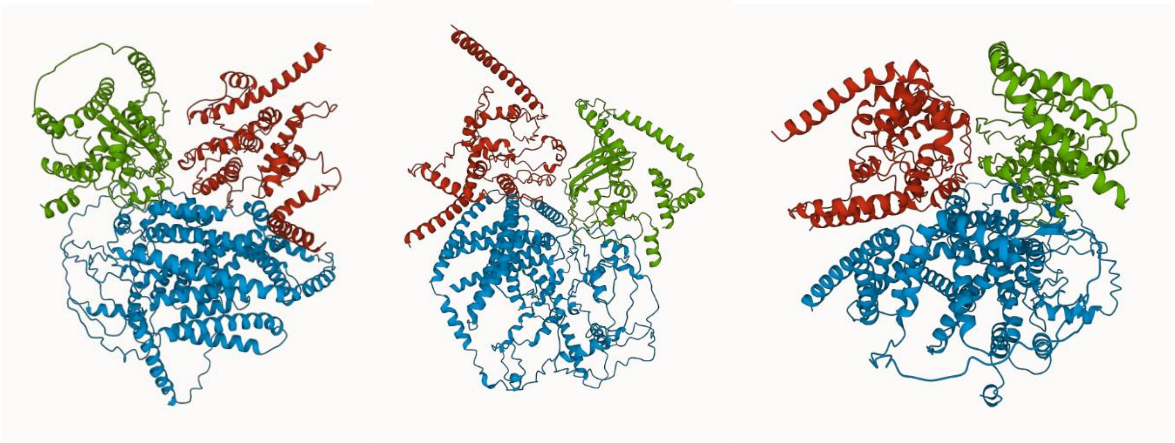

Figure 31 AlphaFold2 *Donax trunculus* prediction of ORF1:ORF2:ORF3 complex (three different structures). Blue indicates fragment of ORF1; red ORF2 and green ORF3 (only fragment of ORF1 due to the limitation of the modeling software 1400 amino acids in total)

Table 8 Divergence (p-distance) between homologous genes in M-type *Donax* mitogenomes. **A:** nucleotide matrix; **B:** protein matrix

**A:**nucleotide	*D. trunculus vs D. semistriatus*	*D. trunculus vs D. vittatus*	*D. semistriatus vs D. vittatus*
ORF 1 Long	0.638	0.658	0.430
ORF 2	0.689	0.670	0.428
ORF 3	0.672	0.654	0.414
COX2-3'	0.378	0.367	0.256
5'-COX2	0.516	0.518	0.31
ATP8	0.531	0.544	0.327
ATP6	0.454	0.454	0.25
COX3	0.494	0.519	0.347
COX1	0.460	0.469	0.413
COX1 first 1.5k bp	0.323	0.331	0.264
CYTB	0.376	0.381	0.251
12S rRNA	0.244	0.245	0.109
16S	0.220	0.231	0.101

**B:**protein	*D. trunculus vs D. semistriatus*	*D. trunculus vs D. vittatus*	*D. semistriatus vs D. vittatus*
ORF 1 Long	0.797	0.825	0.526
ORF 2	0.835	0.884	0.538
ORF 3	0.819	0.823	0.532
COX2-3'	0.355	0.348	0.206
5'-COX2	0.550	0.547	0.339
ATP8	0.667	0.667	0.313
ATP6	0.541	0.515	0.203
COX3	0.542	0.553	0.327
COX1	0.471	0.479	0.406
COX1 first 1.5k bp	0.239	0.247	0.147
CYTB	0.352	0.369	0.191

Table 9 Divergence (p-distance) between *nadh* genes from F-type mitogenomes and ORFs from M-type *Donax* mitogenomes (nucleotide matrix).

D. trunculus Nucleotide	p-distance ORF1 long	p-distance ORF2	p-distance ORF3
NAD1	0.613	0.669	0.699
NAD2	0.658	0.683	0.695
NAD3	0.611	0.634	0.669
NAD4	0.653	0.664	0.664
NAD4L	0.635	0.653	0.639
NAD5	0.657	0.668	0.666
NAD6	0.656	0.679	0.668

D. semistriatus Nucleotide	p-distance ORF1 long	p-distance ORF2	p-distance ORF3
NAD1	0.665	0.678	0.636
NAD2	0.676	0.669	0.647
NAD3	0.634	0.631	0.653
NAD4	0.694	0.664	0.662
NAD4L	0.628	0.691	0.660
NAD5	0.669	0.661	0.657
NAD6	0.658	0.668	0.682

D. vittatus Nucleotide	p-distance ORF1 long	p-distance ORF2	p-distance ORF3
NAD1	0.666	0.663	0.655
NAD2	0.617	0.668	0.654
NAD3	0.656	0.634	0.642
NAD4	0.633	0.648	0.667
NAD4L	0.607	0.656	0.601
NAD5	0.652	0.645	0.664
NAD6	0.661	0.651	0.686

Table 10 Divergence (p-distance) between *nadh* genes from F-type mitogenomes and ORFs from M-type *Donax* mitogenomes (protein matrix).

D. trunculus Prot	p-distance ORF1 long	p-distance ORF2	p-distance ORF3
NAD1	0.758	0.856	0.872
NAD2	0.798	0.833	0.882
NAD3	0.737	0.775	0.850
NAD4	0.803	0.814	0.802
NAD4L	0.768	0.840	0.768
NAD5	0.808	0.825	0.824
NAD6	0.774	0.863	0.804

D. semistriatus Prot	p-distance ORF1 long	p-distance ORF2	p-distance ORF3
NAD1	0.824	0.857	0.802
NAD2	0.840	0.832	0.849
NAD3	0.833	0.817	0.767
NAD4	0.852	0.834	0.833
NAD4L	0.809	0.832	0.779
NAD5	0.857	0.839	0.792
NAD6	0.811	0.791	0.838

D. vittatus Prot	p-distance ORF1 long	p-distance ORF2	p-distance ORF3
NAD1	0.833	0.847	0.830
NAD2	0.779	0.865	0.845
NAD3	0.867	0.808	0.800
NAD4	0.800	0.832	0.796
NAD4L	0.766	0.800	0.684
NAD5	0.847	0.832	0.811
NAD6	0.796	0.817	0.859

Table 11 Divergence (p-distance) between homologous genes in F-type vs M-type *Donax* mitogenomes. A: nucleotide matrix; B: protein matrix.

**A:**Nucleotide	*D. trunculus*	*D. semistriatus*	*D. vittatus*
COX1	0.411	0.407	0.420
COX2	0.513	0.467	0.465
COX3	0.562	0.556	0.540
CYTB	0.416	0.423	0.421
ATP6	0.560	0.544	0.514
ATP8	0.587	0.563	0.500
12S	0.258	0.271	0.241
16S	0.292	0.229	0.194

**B:**Prot	*D. trunculus*	*D. semistriatus*	*D. vittatus*
COX1	0.401	0.392	0.440
COX2	0.565	0.529	0.540
COX3	0.644	0.625	0.664
CYTB	0.401	0.431	0.448
ATP6	0.657	0.652	0.652
ATP8	0.683	0.723	0.634

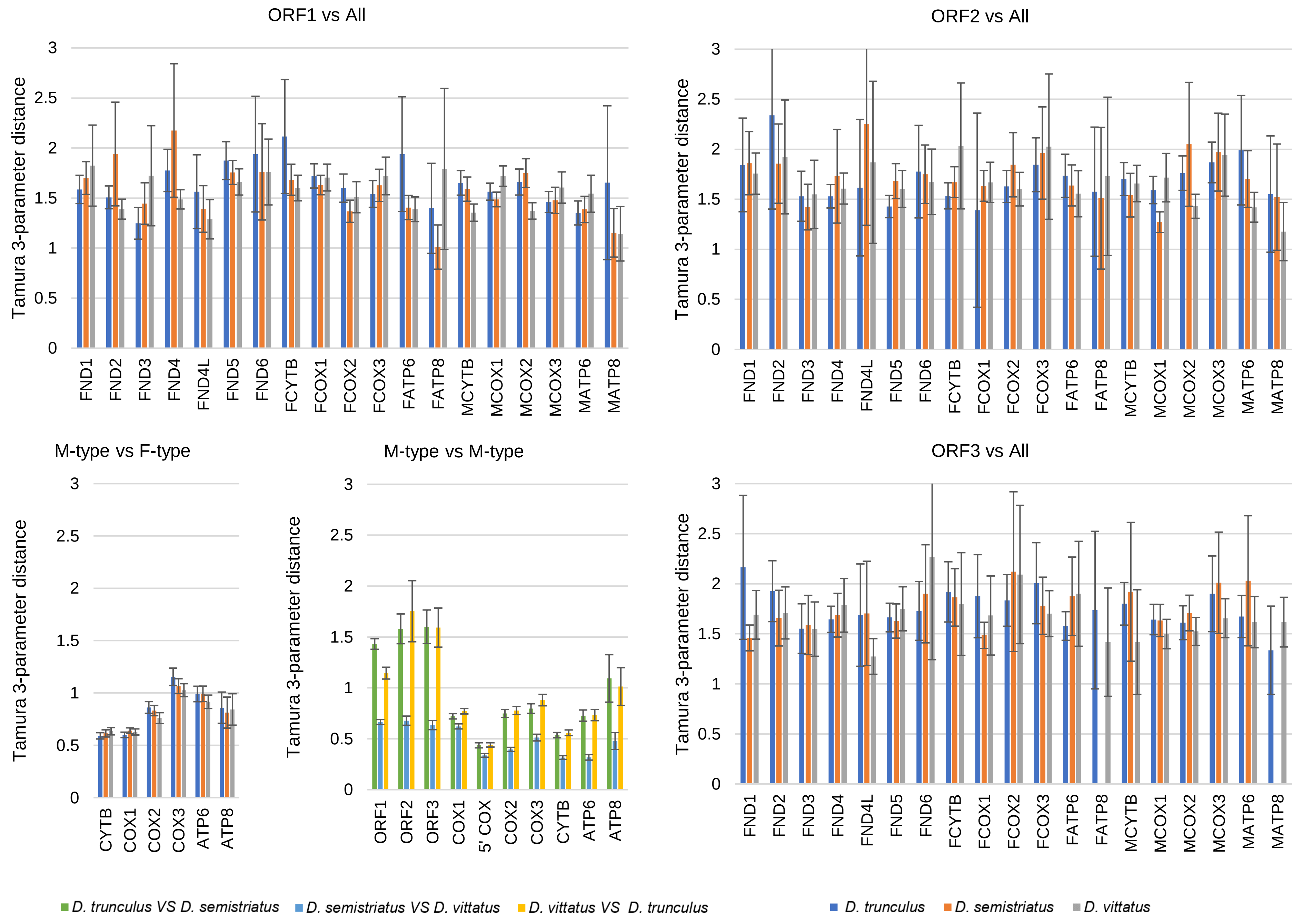

Figure 32. Nucleotide divergence between *Donax* spp, genes calculated with Tamura 3-parameter distance model correcting for G+C-content bias.

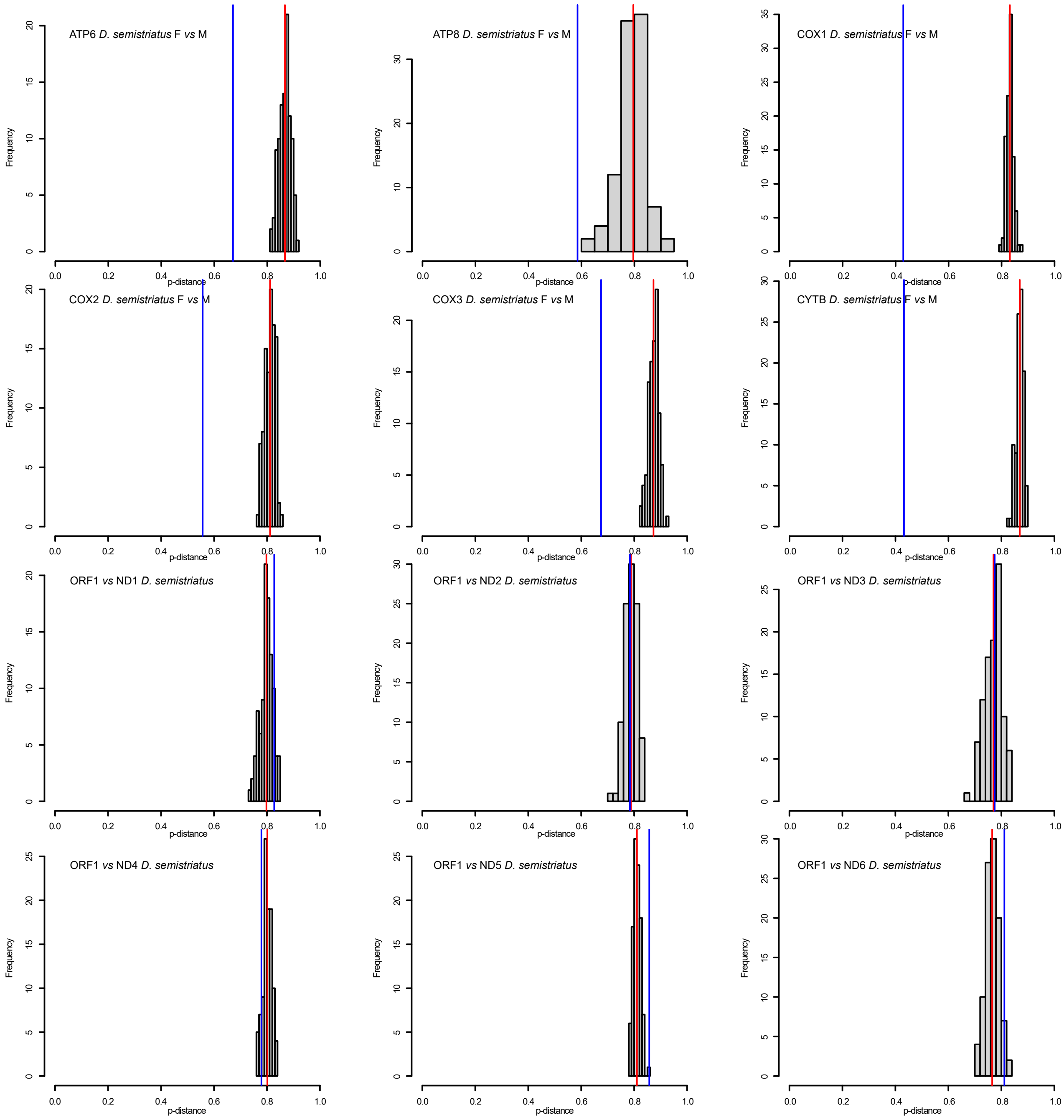

Figure 33. Divergence (p-distance) for pairs of protein sequences (blue line) in relation to 100 randomised amino acid sequences, generated based on compared protein sequences (red line shows average divergence based on 100 randomised sequences). Only data for *Donax semistriatus* is shown.

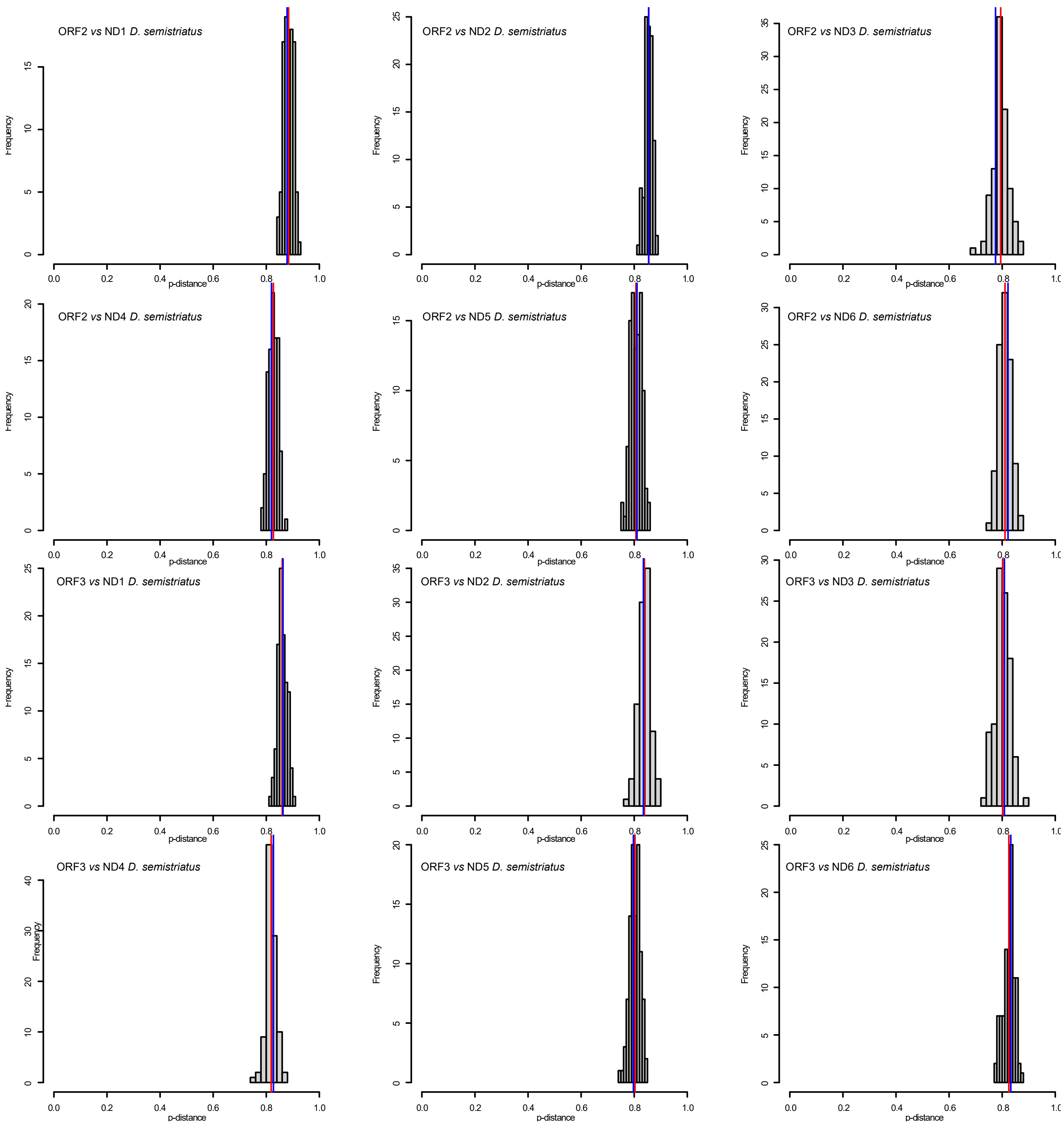

Figure 34 continued. Divergence (p-distance) for pairs of protein sequences (blue line) in relation to 100 randomised amino acid sequences, generated based on compared protein sequences (red line shows average divergence based on 100 randomised sequences). Only data for *Donax semistriatus* is shown.
